# ß1 Integrin Binding Phosphorylates Ezrin at T567 to Activate a Lipid Raft Signalsome Driving Invadopodia Activity and Invasion

**DOI:** 10.1371/journal.pone.0075113

**Published:** 2013-09-24

**Authors:** Ester Antelmi, Rosa A. Cardone, Maria R. Greco, Rosa Rubino, Francesca Di Sole, Nicola A. Martino, Valeria Casavola, MariaLuisa Carcangiu, Loredana Moro, Stephan J. Reshkin

**Affiliations:** 1 Department of Bioscience, Biotechnology and Biopharmacologics, University of Bari, Bari, Italy; 2 Department of Pathology, Anatomic Pathology A Unit, Istituto Nazionale Tumori, Milan, Italy; 3 Department of Medicine, University of Maryland School of Medicine and the Medical Service, Department of Veterans Affairs Medical Center, Baltimore, Maryland, United States of America; 4 Department of Animal Production, Faculty of Biotechnological Sciences, University of Bari, Bari, Italy; 5 Institute of Biomembranes and Bioenergetics (IBBE), CNR, Bari, Italy; University of Birmingham, United Kingdom

## Abstract

Extracellular matrix (ECM) degradation is a critical process in tumor cell invasion and requires matrix degrading protrusions called invadopodia. The Na^+^/H^+^ exchanger (NHE1) has recently been shown to be fundamental in the regulation of invadopodia actin cytoskeleton dynamics and activity. However, the structural link between the invadopodia cytoskeleton and NHE1 is still unknown. A candidate could be ezrin, a linker between the NHE1 and the actin cytoskeleton known to play a pivotal role in invasion and metastasis. However, the mechanistic basis for its role remains unknown. Here, we demonstrate that ezrin phosphorylated at T567 is highly overexpressed in the membrane of human breast tumors and positively associated with invasive growth and HER2 overexpression. Further, in the metastatic cell line, MDA-MB-231, p-ezrin was almost exclusively expressed in invadopodia lipid rafts where it co-localized in a functional complex with NHE1, EGFR, ß1-integrin and phosphorylated-NHERF1. Manipulation by mutation of ezrins T567 phosphorylation state and/or PIP2 binding capacity or of NHE1s binding to ezrin or PIP2 demonstrated that p-ezrin expression and binding to PIP2 are required for invadopodia-mediated ECM degradation and invasion and identified NHE1 as the membrane protein that p-ezrin regulates to induce invadopodia formation and activity.

## Introduction

The dissemination of metastasis is the leading cause of cancer fatality, underlying the need for new therapeutic approaches specifically focusing on tumor cell spreading to distant sites [1]. Despite considerable progress in our understanding of cancer biology, leading to discovery of novel biological treatments, limitations in our understanding of invasion/metastasis has limited development of anti-metastatic therapies [2]. The invasive process requires changes in tumor cell adhesion properties, cell motility and proteolytic remodeling of the extracellular matrix (ECM). It is now well established that aggressive tumor cells have a specific structure dedicated to promoting their increased invasive ability: the Beta1 (ß1)-integrin activated, protease and actin rich plasma membrane structures called invadopodia, that play a central role in driving cancer cell dissemination in the body through the directed proteolysis of the ECM [3-7]. Indeed, the amount of matrix degradation associated with invadopodia activity has been shown to directly correlate with invasive potential making it crucial to understand their dynamics in order to design efficient treatments against metastasis.

However, the complex molecular mechanisms governing the formation and function of invadopodia are still not well understood making the elucidation of the basic mechanisms of invadopodia-driven tumor invasion a major challenge in tumor biology. While there have been many studies looking at the proteins that regulate cytoskeletal organization such as Arp 2/3, WASP, cortactin and the small G-protein Cdc-42 [8 for review], there has yet been no study concerning how the cytoskeletal actin core of the invadopodia interacts with and is bound to the membrane of the invadopodia. In this respect, we still know fairly little concerning the interplay of biochemistry and cell structure that underlies their development and function [3,5,6].

In this respect, recent advances have revealed that the Na^+^/H^+^ exchanger type 1 (NHE1) is localized at invadopodia and its activity has a double function in driving both ß1 integrin-stimulated invadopodia formation and their proteolytic activity through (i) the acidification of the extracellular peri-invadopodia nanospace which is necessary for ECM proteolysis [9] and (ii) the alkalinization of the invadopodia cytosol which causes the release of cofilin from cortactin to stimulate the dynamic process of invadopodia protrusion [10]. Interestingly, both EGF [9,10] and tumor hypoxia [11] enhance cancer cell invasiveness through invadopodia formation by promoting NHE1 activity.

The structural mechanisms by which NHE1 and the actin cytoskeleton in invadopodia are functionally interconnected has remained undefined. In the above papers, it was hypothesized that members of the ERM (ezrin, radixin, moesin) family of adapter proteins could be the probable physical linkers of the NHE1 to the invadopodia actin cytoskeleton since one of the members, ezrin, has been shown to bind to both NHE1 and F-actin and to participate in their reciprocal regulation [12-14]. Ezrin contains an N-terminal domain that recruits a variety of membrane receptors/transporters and a C-terminal domain termed the COOH-terminal ERM-associated domain (C-ERMAD) that binds to the actin cytoskeleton, characteristics which create functional complexes that regulate many processes [15,16], including cell proliferation, survival and apoptosis [17], migration [17,18], spatiotemporal control of various signaling molecules including cAMP [19] and the formation of microvilli [20-22].

In line with these functions, recent studies have identified a central role for ezrin in multiple malignancies. It has been reported to be overexpressed in diverse cancers where it correlates with aggressive stage and poor prognosis [23-40]. A recent paper reported that ezrin overexpressing rectal tumors had a shorter time to local recurrences [41] (Jörgren et al., 2012). In line with these patient data, experimental manipulation of ezrin expression with siRNA or overexpression decreases or increases, respectively, motility and/or invasion in a variety of cell lines [42-52].

Interestingly, a shift from apical membrane to cytoplasmic expression of ezrin has been associated with dedifferentiation, invasiveness, and poor prognosis in colorectal [53], head & neck [54] and breast cancer [55,56]. This suggests that abnormal cellular localization of ezrin may lead to the deregulation of several functions in tumor cells that lead to metastasis, including the acquisition of an invasive phenotype and further suggests that a probable post-translational modification of ezrin is taking place as cancer progresses. In this respect, it is well known that ezrin is activated by conformational changes triggered by a sequential binding of ezrin N-terminal FERM (band 4.1, ERM) domain to phosphatidylinositol 4,5-biphosphate (PIP2) followed by phosphorylation of a conserved threonine residue (T567) in its C-terminal domain [57,58]. The phosphorylation step stabilizes ezrin binding to the actin cytoskeleton and restricts its binding to specialized membrane domains [15] where the active ezrin assembles and integrates signaling molecules to exert diverse downstream effects [58]. Importantly, while some of the functional studies observed that it was the phosphorylated form of ezrin to drive metastasis [42-45,47,48,51], the underlying mechanism is still unknown.

Altogether, these studies demonstrate that not only the expression level of ezrin but also its phosphorylation status and subcellular localization should be considered to better understand its role in tumor progression. However, in breast cancer information on the relationships between ezrin, p-ezrin and clinical-pathological tumor characteristics are lacking. Furthermore, while the above studies suggest that ezrin plays a role in breast cancer progression, neither its specific mechanism(s) of action in driving cancer nor the signal transduction systems involved in its contribution to the metastatic spreading of breast tumors have so far been described. The aim of this study was to provide a bridge between *in vivo* and *in vitro* studies to better understand the role and dynamics of p-ezrin in metastasis.

Here, we observe that p(T567) ezrin is overexpressed in the membrane in aggressive human breast tumors and in the invadopodia in invasive breast cancer cells where it forms protein-protein signaling complexes with NHE1, ß1-integrin, EGFR and phosphorylated NHERF1. Further, p-ezrin enhances NHE1 activity at invadopodia, invadopodial-dependent ECM proteolysis and cell invasion, thus inducing an invasive phenotype in breast cancer cells *in vitro* by coordinating an ECM proteolytic/invasion signal module. We hypothesize that binding to the ECM through the ß1-integrin receptor locally induces ezrin phosphorylation at T567 which promotes the NHE1 signal complex activating it and leading to (i) an altered cytoskeletal organization and the development of the invadopodia [9,10] and (ii) an increased NHE1-dependent H^+^-efflux with its consequent matrix proteolysis [9]. The present study adds ezrin in its T567 phosphorylated state as an important player in the understanding the molecular mechanisms behind the cancer invasive process. We believe that p-ezrin could serve as a marker potentially applicable to the detection and identification of pre-symptomatic cancers and, secondly, could be exploited as a therapeutic target in those cancers.

## Results

### Phospho(pT567)-ezrin expression is associated with more aggressive metastatic progression, poor prognosis and HER-2 expression

**Figure 1:**
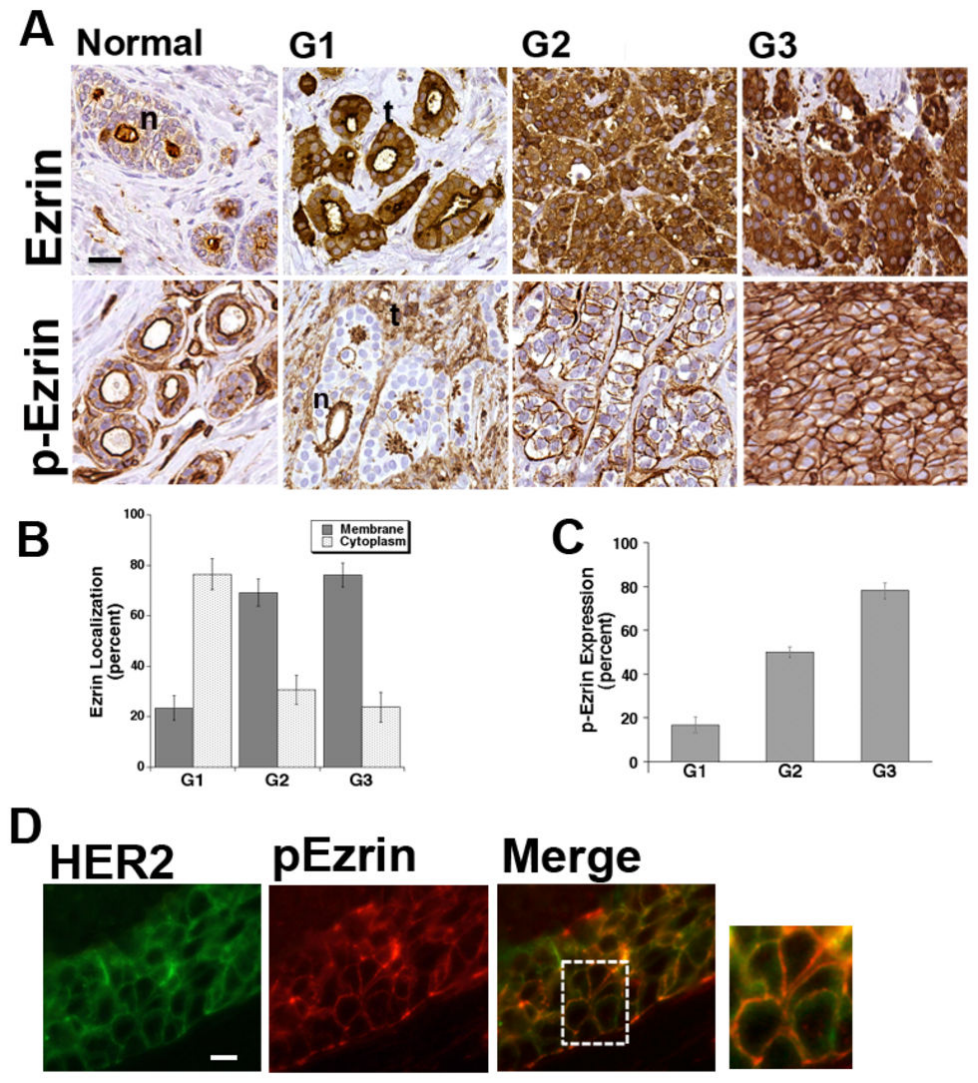
Analysis of ezrin and p-(T567) ezrin expression and localization in human normal and breast cancer. (A) representative immunolocalization (IHC) of total ezrin and p-ezrin in a normal breast and in breast tumor sections of increasing Grade from left to right panels, bar equals 10µm. The increased ezrin and p-ezrin expression in the large disorganized, breast tumor lobules (t) displays diffuse distribution in the cells of the lobule while normal, organized breast lobules (n) have a strictly apical distribution. (B) Histogram showing that total ezrin in tumor samples increases its relative membrane localization as Grade increases. (C) Histogram showing that relative p-ezrin expression in tumor samples increases as Grade increases. (D) Immunofluorescence was directly performed for HER2 (green) and p-ezrin (red) in a breast tumor section. The merge and the magnification of the area in the white square shows that there was a high amount of co-localization of the two proteins in the membrane. bar = 10µm.

To investigate the role of ezrin and p(T567)-ezrin in breast cancer invasion, we assessed their expression and distribution in human breast cancer tissues. We first asked whether total and phospho-ezrin are overexpressed in human breast tumors and how their relative localization may change with progression. Relative total and phospho-ezrin protein expression was measured with Immunohistochemistry (IHC). Figure 1A shows typical IHC staining for ezrin and p-ezrin in tissues derived from a normal breast or from tumors of different grades. As recently reported [59], in the normal lobule total ezrin is highly localized in and limited to the apical membrane while in the lower grade tumors total ezrin is heavily localized in the cytosol and shifts to a more plasma membrane localization as the the tumor advances. New to this study, p-ezrin was always localized to the plasma membrane. A case study of 75 consecutive subjects (Figure 1B), verified that total ezrin increased its plasma membrane localization as the tumors progressed and, importantly, that the relative amount of p-ezrin strongly increased as the tumor grade increased (Figure 1C) while that of total ezrin did not change (data not shown).

We then correlated p-ezrin protein expression measured as above in primary breast tumors with respect to patient clinico-pathological disease characteristics such as age, tumor size, proliferation index (Ki67), presence of regional nodal metastases (Lymph Node), and Progesterone receptor status, tumor cytohistological de-differentiation (Grade), HER2 receptor status and Nottingham Prognostic Index (NPI) (Table 1). Overall p-ezrin protein expression significantly increased with increasing age, tumor cytohistological de-differentiation (Grade), proliferation (Ki-67) and with a poorer prognosis as indicated by an increasing Nottingham Prognostic Index (NPI) [60]. Correlation analysis further revealed that tumor p-ezrin protein expression levels were positively correlated with increasing levels of the proto-oncogene receptor HER2/ErbB2 [61,62]. This association with HER2 was also observed in an immunofluorescence analysis of a histological section from a patient in which an architecturally disorganized, tumor lobule strongly co-expressed HER2 (green) and p-ezrin (red) at the membrane (Figure 1D).

**Table 1:** Association of pEzrin expression with clinico-pathological parameters.

	pEzrin -		pEzrin+		Tot=75	
	n	%	n	%	tot	P value
Age						
30-59	24	70	10	30	34	0.034
60-90	19	46	22	54	41	
Grade						
I	20	83	3	17	24	0.0056
II	14	50	14	50	28	
III	9	39	14	61	23	
Lymph node						
Negative	33	62	20	38	53	NS
Positive	10	45.5	12	54.5	22	
Dimension						
Negative	22	58	16	42	38	NS
Positive	21	57	16	43	37	
PgR						
Negative	8	44.5	10	55.5	18	NS
Positive	34	61	22	39	56	
HER2						
Negative	40	63.5	23	36.5	63	0.013
Positive	3	25	9	75	12	
Ki-67						
Negative	34	65	18	35	52	0.012
Positive	7	33	14	67	21	
NPI						
GPG (<2.5)	25	67	12	33	37	0.048
MPG (2.5-3.5)	13	59	9	41	22	
PPG (>4.5)	5	31	11	69	16	

Phospho(T567)-ezrin (p-ezrin) expression was measured in IHC; n = 75 breast carcinomas (median age 60). Significance between median p-ezrin expression values for Grade and NPI were evaluated by the Kruskal-Wallis non-parametric ANOVA test while the Mann-Whitney non-parametric test was applied to age, size, Ki-67, node status and receptor status. NPI: Nottingham Prognostic Index. GPG: Good Prognostic Group; MPG: Medium Prognostic Group; PPG: Poor Prognostic Group.

### Phospho (T567)-ezrin is enriched in invadopodia and co-localizes with cortactin, NHE1 and ß1-integrin

While these clinical data indicate that plasma membrane p-ezrin expression is related overall to a more aggressive phenotype of breast cancer, the mechanism(s) involved in its role as a metastasis promoter are unknown. We were particularly intrigued by the strong correlation with HER2, suggesting that p-ezrin expression could be related to invasive properties, which plays a critical role in driving metastatic progression [63]. Therefore, we next went on to determine its expression, interaction with other proteins in invadopodia, the invasive organ of metastatic cells, and its role in invasion.

**Figure 2:**
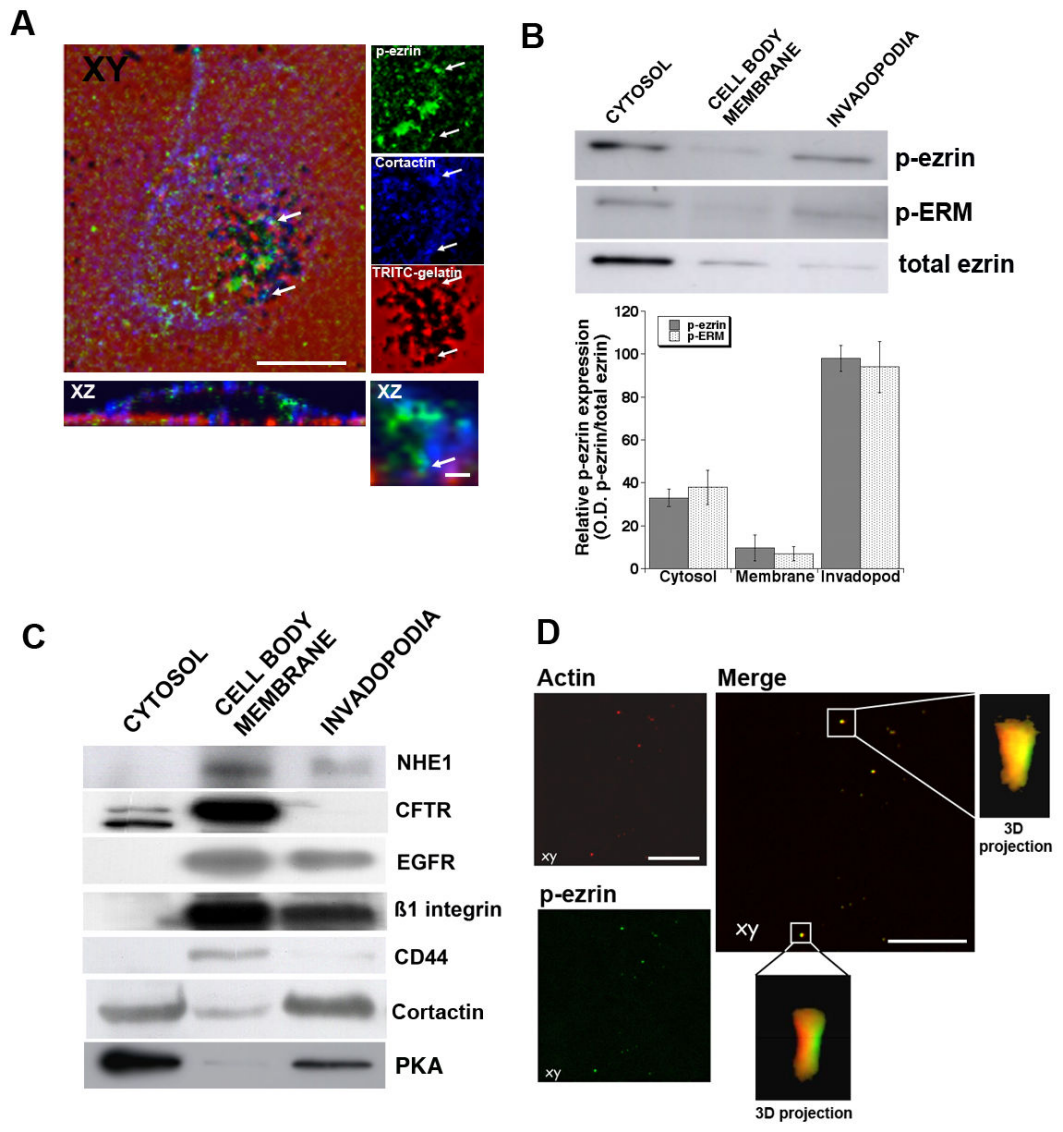
p-(T567) ezrin is preferentially expressed in invadopodia. Invadopodia protein expression on 2% cross-linked gelatin layers. (A) Confocal immunofluorescence micrograph of MDA-MB-231 cells cultured on cross-linked rhodamine (TRITC)-conjugated gelatin. Matrix degradation is visualized as loss of red fluorescence background and cells were stained with anti-cortactin antibody (blue) and anti-p-ezrin antibody (green). There was widespread, focalized zones of degradation and cortactin and p-ezrin were both localized and often co-localized in digested areas as indicated by white arrow in both representative axial (XY) and sagittal (XZ) sections. Representitive IF of 3 independent experiments. Scale bars = 10 µm. (B & C) To better visualize invadopodia entrapped in the ECM, the cell bodies of cells incubated for 6 hr on a layer of 2% cross-linked gelatin were removed and separated into cytosol and plasma membrane while entrapped invadopodia were extracted from the gelatin as described in Methods. Proteins of the three fractions were assayed in Western Blot analysis. The upper panel of (B) shows the expression of p-ezrin with a specific antibody and with an antibody for all the forms of phosphorylated ERM proteins and an antibody for total, non-phosphorylated ezrin, while the lower panel displays a histogram of the relative expression of p-ezrin or p-ERM to total ezrin in the three fractions, n= 12. p<0.001 with respect to expression in the invadopodia. (C) expression of a series of proteins to determine their relative expression in the fractions. (D) Using the above scraping protocol on coverslips, immunofluorescence was directly performed for p-ezrin (green) and actin (red) in the invadopodia that remained in the cross-linked gelatin. Representitive IF of 4 independent experiments. Axial (XY) and sagittal (XZ) sections show a high co-localization of p-ezrin and actin in invadopodia. Scale bars = 2 µm.

As a starting point of the study of the p-ezrin regulation of focal invadopodial-dependent ECM degradation, we measured the association of total and p-ezrin expression with focal proteolytic ECM degradation and with the expression of the actin nucleation factor and invadopodia marker, cortactin. We first utilized the classic technique in which cells are plated on top of a chemically cross-linked layer of 2% porcine gelatin labeled with rhodamine and focal digestion is observed as the removal of background red substrate fluorescence. As shown in Figure 2A, cells of the highly invasive human breast cancer line, MDA-MB-231, incubated on this gelatin overnight, removed focal zones of gelatin observed as non-fluorescent areas both under the cell body and at the cell periphery. Confocal immunofluorescence analysis of the expression of the invadopodial marker, cortactin (blue) and endogenous p-ezrin (green) showed that these two proteins were associated with areas of focal digestion and, further, were often colocalized (see white arrowheads for examples) in various focal digestive zones.

To obtain a quantitative comparison of the distribution of various proteins in invadopodia, cells plated as above on 2% cross-linked gelatin were fractionated for cytosol, cell membrane and invadopodia fractions as previously described [9] assayed by Western Blotting for total ezrin, p-ezrin and p-ERM (Figure 2B, upper panel) and for NHE1, CFTR, cortactin, Protein Kinase A (PKA) and the receptors EGFR, ß1-integrin and CD44 (Figure 2C). These Western Blot measurements in the three fractions showed that p-ezrin, p-ERM and cortactin were all very over-expressed in the invadopodial fraction compared to the membrane fraction. An analysis of p-ezrin and p-ERM expression standardized to total ezrin expression (Figure 2B, lower panel) showed that in invadopodia almost all of the ezrin present was phosphorylated at T567 while a very small fraction of ezrin was phosphorylated in the membranes of the rest of the cell. As gelatin principally activates ß1 integrin, we stimulated the cells with the ß1 integrin activating antibody, P4G11, to determine if the phosphorylation of ezrin at T567 was a direct consequence or not of the activation of this receptor. As can be seen in Figure S1A, the specific activation of the ß1 integrin results in a rapid increase in p-ezrin that was maximal at 24 hours. Interestingly, Figure 2C shows that, of the three receptors assayed, ß1 integrin and EGFR were expressed in both the membrane and invadopodia fractions while CD44 was found to be expressed almost exclusively in the membrane fraction. This primarily membrane distribution of CD44 was verified in confocal analyses of cells plated on Matrigel containing DQ-BSA (Figure S2).

To better visualize invadopodia entrapped in the ECM, we removed the cell body of cells incubated for 6 hr on this 2% cross-linked porcine gelatin as previously described [9] and then directly performed confocal immunofluorescence for actin and p-ezrin expression in the invadopodia that remained inside the gelatin (Figure 2D). In confocal immunofluorescence inside the gelatin layer, p-ezrin and actin were observed to co-localize in various groups of invadopodia and Z-analysis showed that the invadopodia were approximately a micron in width and ranged from 3 to 5µm in length consistent with the size and form of invadopodia reported in studies utilizing combined confocal and electron microscopy [64]. Statistical analysis of their co-localization by the calculation of the Li’s Intensity Correlation Quotient (ICQ) of the variance in the distribution of the two proteins, demonstrated that actin and p-ezrin localization at invadopodia were highly co-dependent (ICQ=0.427 ± 0.035, n = 3, p<0.001).

Since the localization of invadopodial activity by the removal of entrapped fluorescence in the ECM does not permit a direct analysis of the proteins associated with proteolytic activity, we next used a modified protocol that we developed based on the degradation-dependent release of fluorescence of a quenched fluorophore (DQGreen-BSA) that has been dissolved in a thicker, non cross-linked matrix layer comprised either of a single ECM component such as collagen I or of the natural, complex mixture found in Matrigel [9]. In this protocol, digestion is observed as the appearance of unquenched fluorescence in a dark background rather than as the removal of high background fluorescence. Importantly, this protocol also gives a more sensitive measure of the initiation and/or low levels of digestion and, further, permits the quantification of local proteolytic activity, the high-resolution mapping of individual focal cleavage sites and a more exact confocal co-localization between focal digestion and proteins of interest. Preliminary three color fluorescence confocal microscopy experiments in cells cultured on DQGreen-BSA Matrigel for 6 hrs demonstrated that, indeed, actin was co-localized with cortactin in the areas of observed focal proteolysis of ECM bound DQGreen-BSA in a highly significant manner in Matrigel (data not shown). These properties of the actin/cortactin punctate digestive structures are clearly consistent with that of invadopodia reported previously [3-5] and ‘invadopodial’ structures formed in Matrigel by the same cell line [9].

**Figure 3:**
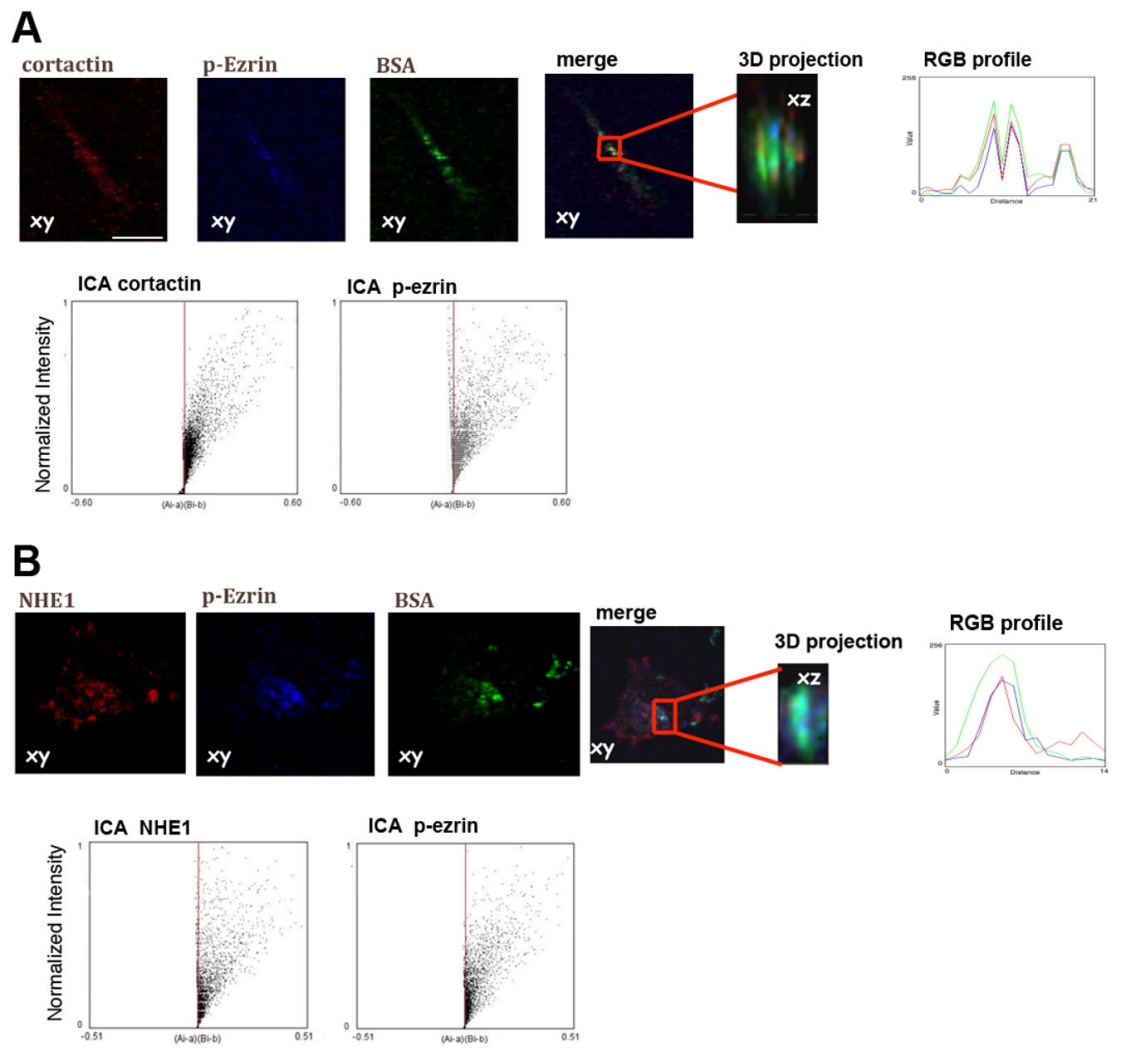
p-ezrin is expressed and co-localized with NHE1 in invadopodia of cells on Matrigel. To better visualize invadopodial focal digestion and protein localization in Matrigel, we developed an *in* *situ* zymography technique using the quenched fluorescent substrate, DQ-Green-BSA, such that a quantifiable fluorescence is released only upon digestion of the matrix. Cells seeded on Matrigel were allowed to digest the fluorigenic substrate and after fixation cells were assayed in immunofluorescence. The images show (A) cortactin and p-ezrin and (B) NHE1 and p-ezrin immunolocalization at BSA proteolytic spots (green) in axial planes taken at the bottom of the cells (XY). For each of the two fields, XZ zoomed sections of the above representative regions of interest (red box) are shown at the side. As can be seen in green, low levels of basal, diffuse digestion were observed under and around the cells together with more restricted areas of high levels of focal digestion. Representative IFs of 5 independent experiments. Scale bar = 10 µm. Right panels: XZ plane and relative magnification of indicated area show the high co-localization of cortactin and NHE1 with p-ezrin and proteolysis in RGB analysis. Lower panels: ICA analysis plots using the JACoP image analysis plugin in ImageJ of cortactin/p-ezrin and NHE1/p-ezrin shows their highly significant co-localization. Average ICQ values for all the experiments are presented in the text.

Identical experiments demonstrated that p-ezrin was co-localized with cortactin in the areas of observed focal proteolysis of ECM bound DQGreen-BSA in a highly significant manner in Matrigel (Figure 3A). Therefore, we next examined the association of p-ezrin and NHE1 (Figure 3B) expression with ECM focal digestion in three color fluorescence confocal microscopy in cells cultured on DQGreen-BSA-Matrigel for 6 hours and observed that NHE1 highly co-localized with both p-ezrin and focal ECM proteolysis both along the edge of the cell as well as at the tip of leading edge pseudopodia. The size of these p-ezrin/cortactin and p-ezrin/NHE1 expressing proteolytic, invadopodial structures were very similar to those observed above in Figure 2 and Z sectioning revealed that these structures protruded vertically from the ventral membrane into the Matrigel substrate at sites of focal ECM degradation with a size of the proteolytic structures (invadopodia) very similar to those observed above on crossed-linked gelatin: approximately 1 µm in diameter and 3-5 µm in length. RGB analysis of the Z-sections showed that the indeed actin colocalization occurred in areas of focal digestion. Furthermore, Intensity Correlation Analysis (ICA, panels under images) and the Li’s Intensity Correlation Quotient (ICQ) of the Z-sections revealed a high co-dependence between the distribution of p-ezrin and cortactin or NHE1 (ICQ = 0.381 ± 0.015, n = 3, p < 0.001 and 0.403 ± 0.21, n = 3, p < 0.001 for the p-ezrin/cortactin and p-ezrin/NHE1, respectively).

### p-ezrin binds NHE1 and ß1 integrin/EGFR in a ternary complex in tumor cells

**Figure 4:**
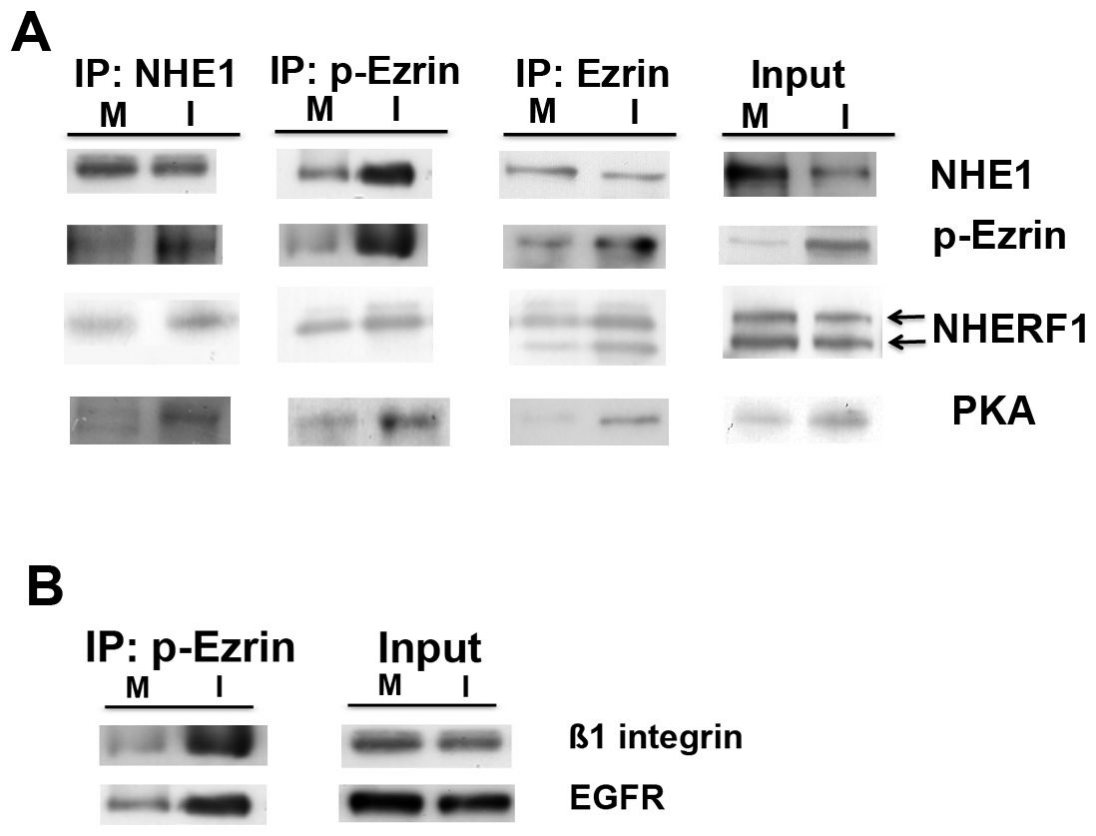
p-ezrin binds NHE1, p-NHERF1, PKA and ß1 integrin/EGFR in a ternary complex in tumor cells. (A) MDA-MB-231 cells were seeded for 24 hr on 2% gelatin and cytosol, cell body membrane and invadopodia fractions were separated as above. Each cell membrane or invadopodia fraction was then immunoprecipitated with anti-NHE1, anti-p-ezrin or anti-ezrin and the precipitated immunocomplex was probed for the expression of NHE1, p-ezrin, NHERF1 and RIIß subunit of PKA by Western Blot. (B) the fractions were immunoprecipitated with anti-p-ezrin and the precipitated immunocomplex was probed for the expression of ß1 integrin and EGF receptors by Western Blot. Protein input for each protein was measured by Western Blot of the total input with the same antibodies. Representitive blots of 4 independent experiments.

The fractionational and confocal co-localization suggested the possibility that p-ezrin could be directly involved in the regulation of NHE1 activity in the tumor cells. Ezrin and p-ezrin scaffolding function is mediated by its binding to other proteins via its N-terminal domain (N-ERMAND) and NHE1 is a known partner of ezrin [12-14]. To analyze this possible physical/biochemical association between p-ezrin and NHE1 and identify other members of this protein-protein complex, we conducted co-immunoprecipitation (co-IP) experiments and immunoblot analysis for each of the putative complexed proteins with anti-ezrin, anti-p-ezrin and anti-NHE1 in the membrane (m) and invadopodia (i) fractions isolated as above from cells plated on 2% gelatin (Figure 4A). We utilized aliquots from each fraction to measure the input expression of the three proteins in each fraction and the self-identification of each protein in the immunocomplex to verify the success of the immunoprecipitation. While the ‘input’ NHE1 was more expressed in the membrane (M) fraction, the antibody against p-ezrin precipitated NHE1 much more strongly in the invadopodia fraction (I) that in the membrane fraction (M) and the antibody against total ezrin only weakly precipitated NHE1 in either fraction. The antibodies against all three proteins much more strongly precipated p-ezrin in the invadopodia fraction, reflecting its proportional expression levels. Reciprocially, the amount of p-ezrin bound to the NHE1- and total ezrin- immunocomplexes was much stronger in the invadopodia fraction than in the membrance fraction. These data demonstrate that p-ezrin directly binds to NHE1 in the invadopodia and support the confocal IF experiments showing their co-localization in the invadopodia (Figure 3B).

The organization of discrete, functional macromolecular signaling complexes is now known to constitute a critical level of biological activity and their construction is orchestrated by scaffolding proteins containing modular interaction domains that facilitate the association of multiple target proteins. The multifunctional scaffolding protein NHERF1 has been demonstrated to be a major component of signaling complexes and binds to ezrin to regulate membrane dynamics through its c-terminal ERM binding domain [20-22]. Both the non-phosphorylated (approximately 50 kDa) and phosphorylated (approximately 55 kDa) NHERF1 bands were expressed in both the invadopodia and membrane fractions. As shown by the immunocomplexes for each of the three proteins (Figure 4; IP: NHE1; IP: p-ezrin; IP: total ezrin), while the total ezrin immunocomplex precipitated both the 50kDa and 55kDa (phosphorylated) forms of NHERF1, both the p-ezrin- and NHE1- immunocomplexes precipitated only the 55kDa NHERF1 band. These data suggest that it is only phosphorylated NHERF1 that actively participates in the formation of a p-ezrin-NHE1-NHERF1 protein-protein complex and in its regulation of invadopodia function. Ezrin also has an important role as an “A Kinase Anchoring Protein“ (AKAP) in which it binds to the regulatory subunit of Protein Kinase A (PKA) to finely regulate its localization and target proteins. Interestingly, all three immunocomplexes much more strongly precipitated the PKA regulatory II subunit in the invadopodia fraction, reflecting its proportional expression levels.

In order to determine the possible inclusion of the ß1-integrin and EGF receptors in a signaling complex with p-ezrin, we also performed Western Blotting of these proteins in immunocomplexes from the plasma membrane (m) and invadopodia (i) cell fractions of cells that had been plated on 2% gelatin. As seen in Figure 4B, both ß1-integrin and EGF receptors precipitated with the p-ezrin immunocomplex preferentially in the invadopodia fraction, demonstrating a probable direct active involvement of these receptors with the p-ezrin protein-signalling complex. In order to determine the possible direct binding of the ß1-integrin receptor with p-ezrin, we performed two types of experiments. Firstly, we examined the association of p-ezrin and ß1-integrin (Figure S3A) expression with ECM focal digestion in three color fluorescence confocal microscopy in cells cultured on DQGreen-BSA-Matrigel for 6 hours and observed that ß1 integrin highly co-localized with both p-ezrin and focal ECM proteolysis. Secondly, to further analyze the potential direct association between p-ezrin and ß1 integrin at proteolytically active invadopodia, we used an *in situ* Proximity Ligation Assay (*in situ* PLA), which can detect endogenous protein-protein interactions that occur within 40 nm [65] combined with the Matrigel degradation assay (Figure S3B). Indeed, as shown by the red fluorescent staining (upper left panel), p-ezrin associates with ß1 integrin in a large subset of foci of degraded matrix. Interestingly, there are a couple of ‘digestive’ podosome (doughnut shaped structures, white arrows) where proteolysis occurred mostly within the circle. These results demonstrate that some sub-populations of p-ezrin and ß1 integrin closely interact in functionally active invadopodia where they probably both interact with NHE1.

Altogether, these data suggest that stimulation of cancer cells to develop invadopodia by plating on ECM, occurs through the formation of a protein-protein complex formed by NHE1, p-ezrin, ß1-integrin, PKA and p-NHERF1.

### NHE1 and p-ezrin are preferentially associated with lipid rafts in the invadopodia fraction

**Figure 5:**
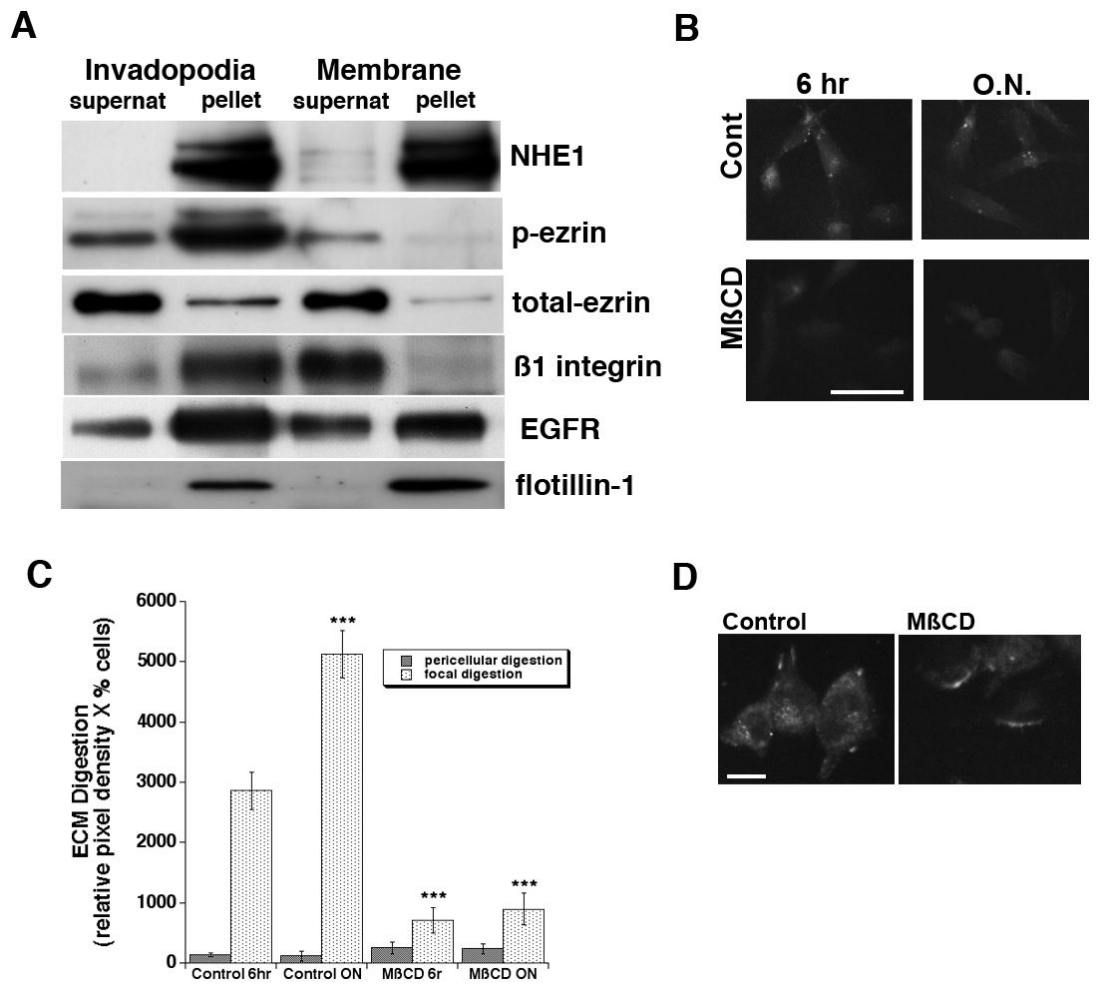
NHE1 and p-ezrin are localized in the same lipid raft fraction in the invadopodia but not in the membrane compartments and lipid raft cholesterol is necessary for invadopodia function. (A) Raft (pellet) and nonraft (supernatant, Supernat) fractions were separated from the above membrane and invadopodia fractions by incubating the fractions (100 µg protein) at 4°C with lysis buffer containing 1% of Lubrol. Insoluble material was collected into pellets (centrifugation at 100,000 X *g* for 1 hour), and equal amounts of the resuspended pellet (P) and the supernatant (SN) were analyzed by Western Blotting for NHE1, p-ezrin, total ezrin, EGFR and ß1-integrin. Quality of separation was determined by immunoblotting for flotillin-1, a marker for lipid rafts. Representative blots are shown (n = 3). To determine the effect of cholesterol depletion on invadopodia function, MDA-MB-231 cells were incubated for 30 minutes at 37°C in the presence (MβCD-treated) or absence (untreated) of 0.5% MβCD as described in Methods and invadopodia-dependent ECM digestion was analyzed in confocal microscopy for a series of individual cells as described in Methods; (B) typical images of digestion for 6 hrs and overnight in cells treated or not. (C) histogram of a series of experiments performed as in (B). Mean ± S.E.M., n=3, ***p<0.001 for focal proteolysis compared to the control cells. (D) NHE1 immunofluorescence of vehicle or MβCD treated cells demonstrating a change in transporter distribution but not total expression. bar = 10 µm.

Caveolin-1 containing lipid rafts are dynamic plasma membrane platforms that promote specific protein clustering and the formation of functional signalling complexes [66]. Lipid rafts are highly present in invadopodia membranes and are proposed to be required for both invadopodia formation and function [67-70]. Therefore, we further fractionated the membrane and invadopodia isolated fractions into raft (pellet) and nonraft (supernatant, supernat) fractions. As can be seen in Figure 5A, total ezrin and p-ezrin expression were associated with different lipid fractions in invadopodia suggesting that in invadopodia ezrin shifts its localization to lipid rafts when it becomes phosphorylated as has been described in other cell types [50]. Importantly, NHE1 and p-ezrin were preferentially localized in the lipid raft fraction, suggesting that this co-localization of the activated p-ezrin with NHE1 could be involved with their above observed functional interaction. The co-localization of NHE1 and p-ezrin with flotillin-1 clearly shows that these proteins are associated with lipid rafts suggesting that the raft organization is important for the complex formation and invadopodia function. Furthermore, the possible active involvement of the ß1-integrin and EGF receptors in the signaling complex was further supported by their localization in the invadopodial raft fraction (Figure 5A).

To functionally test if the presence of lipid rafts is critical, the cells were subjected to a mild cholesterol depletion with 1 mM methyl-β-cyclodextrin (MßCD) for 1 hr at 37 °C to disrupt lipid rafts and the proteolytic capacity of their invadopodia were measured as above. As can be seen in Figure 5B and C, MßCD treatment reduced proteolytic activity by more than 80% while maintaining the general cell shape and total surface expression of the NHE1 (Figure 5D) demonstrating that a change in transporter distribution but not number probably accounts for the inhibition of ECM proteolytic activity. Taken together, these results suggest that cholesterol depletion has an effect on p-ezrin/NHE1 function and are consistent with previous work demonstrating the importance of lipid rafts in the regulation of NHE1 [71-74]. We propose these interactions occur in caveolin-1-containing lipid rafts in the invadopodial compartment and are responsible for an increased ECM degradative activity.

### Relevance of ezrin T567 phosphorylation and its binding to PIP2 and/or NHE1 in regulating invadopodia proteolytic activity, NHE1 activity and invasion

**Figure 6:**
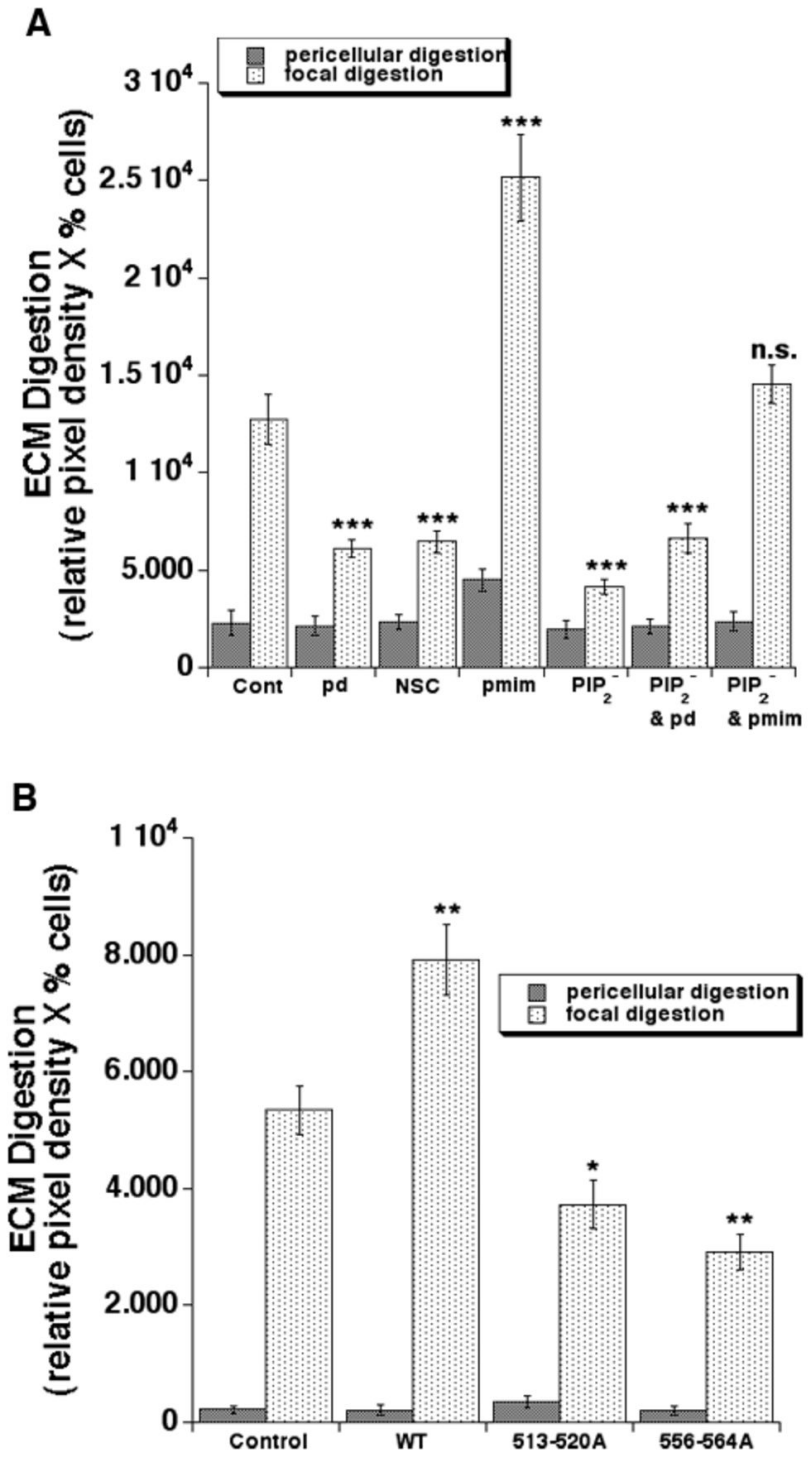
Ezrin phosphorylation and binding to PIP2 and NHE1 binding to both ezrin and PIP2 are necessary for invadopodia proteolytic activity. (A) To examine the role of ezrin T567 phosphorylation and/or binding to PIP2 in invadopodial-dependent focal digestion of the ECM, MDA-MB-231 cells were (A) transfected with empty cDNA (Control) or plasmids contain ezrin cDNA mutated in the T567 site to be either phosphodead (pd) or phosphomimic (pmim) or mutated in the ezrin PIP2 binding site such that it no longer can bind PIP2 (PIP2-) or treated with 1µM of the inhibitor of ezrin T567 phosphorylation, NSC668394 (NSC). (B) To examine the role of NHE1 binding to either ezrin or PIP2 in invadopodia proteolysis, MDA-MB-231 cells were transfected with an NHE1 mutant lacking the ability to bind to ezrin (KR/A 556-564-NHE1-HA) or to bind to PIP2 (KR/A 513-520-NHE1-HA). Two days after transfection, cells were plated on Matrigel with DQ-Green BSA and, 24 hr later, ECM digestion was analyzed in confocal microscopy for a series of individual cells as described in Methods. Mean ± S.E.M., n=4, ***p<0.001 for focal proteolysis compared to the control cells.

We first approached the question of the role of increased endogenous p-ezrin expression in invadopodia by overexpressing constructs of (i) ezrin mutated in the T567 domain to either no longer be able to be phosphorylated (T567A) or to mimic phosphorylation (T567D) together with an ezrin construct that cannot bind to phosphatidylinositol 4,5-bisphosphate (PIP_2_ **^-^**). The mean total invadopodia proteolytic activity of each cell was then assessed by the release of quenched Bodipy fluorescence in Matrigel assays as described in the Methods section (Figure 6A).

The analysis of Matrigel proteolysis revealed that transfection of the MDA-MB-231 cells with the phosphorylation dead T567A ezrin mutant (pd) resulted in an approximately 60% reduction of focal ECM proteolysis while transfection of the phosphorylation mimic T567D mutant (pmim) doubled their total proteolytic capacity. Similar results were obtained when the cells were incubated with 1µM of NSC668394 (NSC, 3rd set of lanes of Figure 6A), a compound that inhibits ezrin phosphorylation at T567 [75]. The activation of ezrin can also occur subsequent to its binding to phosphatidylinositol 4,5-bisphosphate (PIP2), a lipid that is selectively concentrated at the apical surface of polarized epithelia [57,58] and which is also expressed in invadopodia [76]. As can also be seen in Figure 6A, transfection of cells with the ezrin construct in which its binding to PIP2 is abolished (PIP_2_ **^-^**) significantly reduced the total ECM proteolytic capacity to levels similar to the phospho-dead (pd) mutant. Moreover, to assess if both ezrin phosphorylation and its interaction with PIP2 are necessary for invadopodial activity and if they interact, we transfected the cells with double mutants for phosphorylation and PIP2 binding. Importantly, the double negative mutant construct (PIP_2_ **^-^**/pd) did not further reduce ECM proteolytic invadopodial activity, whereas transfection with the double mutant construct containing the negative PIP2 binding mutation together with the phosphorylation mimic mutation (PIP_2_^-^ & pmim) annulled the effect of the two mutations, suggesting that the binding of ezrin with the membrane component PIP2 is also a prerequisite for it regulation of invadopodial activity as previously observed in other cellular systems [57,58] and that they are acting through the same signal cascade. To determine if this phosphorylation of ezrin is part of the ß1 integrin invadopodia signaling network and is necessary for the ß1 integrin stimulation of invadopodia-driven proteolysis, we stimulated cells plated on DQ-labeled Matrigel with 5 mg/ml of an activating antibody against ß1 integrin (P4G11) in the absence or presence of the p-ezrin inhibitor, NSC668394. As can be seen in Figure S1B, in control conditions treatments with the activating antibody stimulated focal proteolysis by approximately 2.4-fold and this stimulation was almost completely abrogated in the presence of the inhibitor.

**Figure 7:**
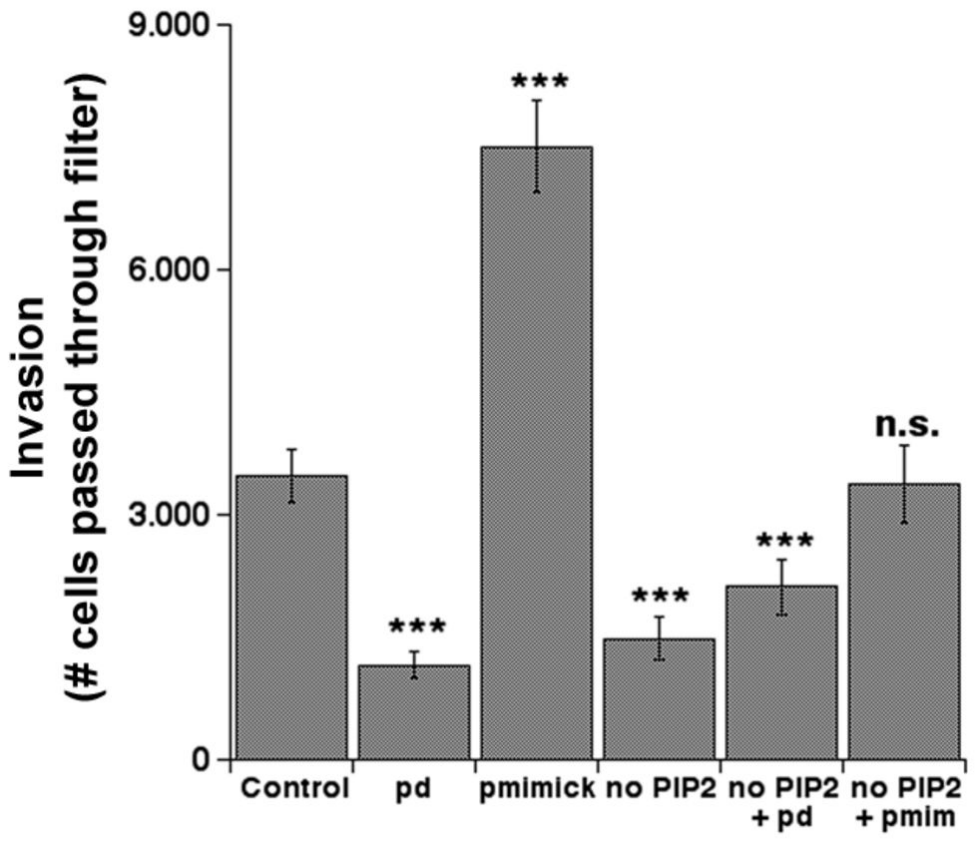
Ezrin phosphorylation and binding to PIP2 are necessary for invasion. To examine the role of ezrin T567 phosphorylation and/or binding to PIP2 in invasive capacity, MDA-MB-231 cells were transfected with empty cDNA (Control) or plasmids containing ezrin cDNA mutated in the T567 site to be either phosphodead (pd) or phosphomimic (pmim) or mutated in the ezrin PIP2 binding site such that it no longer can bind PIP2 (PIP2-). Two days after transfection, a quantitative measure of the degree of *in* *vitro* invasion of MDA-MB-231 cells was measured as the ability to traverse a 8 µm polycarbonate membrane coated with 5 µg matrigel (Chemicon Int., Livermore, CA) as previously described (Cardone et al., 2005). The fluorescent samples were read in a fluorescence plate reader at 480/520 nm (Cary Eclipse Fluorescence Spectrophotometer, Varian). Mean ± S.E., n=5, ***p<0.001 and n.s. is not significant.

On the basis of these data on ECM proteolysis together with reports in various tumor cell lines that p(T567)-ezrin (p-ezrin) drives invasion (see Introduction), we next tested the effect of these mutants on invasion using a 3D invasion assay where the cells must cross a thick layer of Matrigel (Figure 7). Indeed, invasive capacity followed quite closely the pattern of the effect of the ezrin mutants on focal ECM proteolysis, in line with studies suggesting that invadopodia function and p-ezrin are fundamental for cancer cell invasive capacity.

### Relevance of NHE1 binding to ezrin and PIP2 in regulating invadopodia proteolytic activity

If ezrin regulates invasive activity through its modulation of NHE1 activity then we can hypothesize that we will find a similar regulatory pattern of invadopodia ECM proteolysis as we observed above with the ezrin mutants with NHE1 mutants that cannot bind either ezrin (KRA 556-564) or (KRA 513-520) PIP2 (Figure 6B). Indeed, while over-expression of the wild-type (WT) NHE1 construct increased focal digestion by approximately 45%, cells transfected with either the ezrin binding-defective mutant of NHE1 (KRA 556-564) or the PIP2 binding-defective mutant of NHE1 (KRA 513-520) had a greatly reduced amount of focal matrigel digestion compared to cells transfected with the empty vector. While this inhibition is lower than that reported in cells transfected with the ion transport-defective mutant (E266I) of NHE1 [9], it does compare closely with the amount of inhibition observed above with the ezrin mutants.

Altogether these results suggest that both the phosphorylation of ezrin and its interaction with PIP2 are indispensable in controlling invadopodial NHE1 activity and the consequent focal proteolytic capacity, as well as in establishing a compartmentalized functional “signalsome” that allows this activation of invadopodial proteolytic activity. Interestingly, the activity of NHE1 can also be directly regulated by its binding to PIP2 suggesting that the PIP2-dependent regulation of its activity could occur through a dynamic interaction of the two pathways and introduces an additional layer of regulation of invadopodia activity between the cytoskeleton and the membrane.

## Discussion

While a potential role for ezrin in breast tumor progression has recently been demonstrated in immunohistochemical studies that observed an elevated expression and altered localization in breast tumors compared to adjacent normal breast tissue (see Introduction), to date there has not been an evaluation of the clinical-pathological parameters associated with the overexpression of its activated, T567 phosphorylated form (p-ezrin). Here we observe, in a series of breast tumors (Figure 1A-C; Table 1), that while total ezrin changes its localization with progression, p-ezrin remains membrane bound and increases it expression such that it is highly overexpressed in tumors. Further, this overexpression is associated with increasingly aggressive clinical characteristics, with poor prognosis and with increasing HER2 expression, a marker of breast cancer invasion and metastatic progression [61,62]. Altogether, these *in vivo* data predict that p-ezrin would make a novel tumor prognostic marker. This evident and significant increase in p-ezrin expression at the membrane of the more aggressive tumors together with the high co-localization with the oncogene HER2, suggested that p-ezrin expression and function could be related to aggressive behavior. While *in vitro* studies have demonstrated that p-ezrin increases invasion and metastasis, the precise mechanism(s) involved in its role as an invasion promoter are unknown. We were particularly intrigued by the strong correlation with HER2, suggesting that p-ezrin expression could be related to invasion, which plays a critical role in driving metastatic progression. Therefore, we next went on to determine its expression, interaction with other proteins and role in invasion and in invadopodia, the invasive organ of metastatic cells.

Activation of ß1 integrin through integrin-mediated cell substrate adhesion at point contacts constitutes the primary spatial cue leading to the recruitment of ECM-degrading enzymes and formation of a polarized plasma membrane extraflection domain (invadopodia) which penetrates the underlying ECM to produce its focal proteolysis and permit the invasion of the tumor cell [3-7]. Here we present data to demonstrate that binding to the ECM through the ß1-integrin receptor promotes the local phosphorylation of ezrin at T567 which facilitates the formation of a lipid raft localized signalling complex that promotes NHE1-dependent invadopodia formation and proteolytic activity [7,9,10]. Indeed, the specific activation of the ß1 integrin with an activating antibody (P4G11) rapidly phosphorylated ezrin (Figure S1A) and stimulated invadopodia-dependent focal proteolysis that was abrogated by NSC668394, a pharmacological inhibitor of ezrin phosphorylation at T567 (Figure S1B).

As a scaffolding protein, p-ezrin generates spatiotemporal asymmetry at the cell cortex to organize transmembrane proteins with the actin cytoskeleton in-order-to integrate both structure and signaling at the apical junctional complex of polarized epithelial cells which underlies cell polarization and asymmetric cell division [15-18]. In invasive cancer cells, one could predict that the activated form of ezrin, phosphorylated on T567, would increase its relative expression in the organelle dedicated to invasion. Indeed, in Western Blot analysis of isolated fractions we observed that while total ezrin expression was lower in the invadopodia than in the other cell compartments, it was expressed almost entirely in the T567 phosphorylated form while in the rest of the cell the relative level of phosphorylated ezrin was quite low. This preferential invadopodial localization of p-ezrin was confirmed with confocal microscopy on both gelatin and Matrigel and co-localized with NHE1 and ß1-integrin in the invadopodia. These direct interactions were confirmed in co-IP experiments in the isolated fractions where p-ezrin immunocomplexes contained both NHE1 and the ß1-integrin receptor preferentially in the invadopodia.

Caveolin-1 containing lipid rafts, also known as caveolae, are cholesterol- and sphingolipid-enriched dynamic platforms at the plasma membrane which promote specific protein clustering that promotes the formation of functional signaling complexes [66]. These lipid rafts are highly present in the membranes of invadopodia and are proposed to be required for both invadopodia formation and ECM degradative activity [67-70]. Furthermore, in some cancer cells NHE1 has been shown to be localised in caveolin-containing lipid rafts which contributes to regulating its activity and, presumably, its interaction with other proteins [71-74]. Lipid raft fractionation confirmed that in the invadopodia p-ezrin shifts to lipid rafts where it found with the NHE1 together with EGFR and ß1-integrin, suggesting that raft organization is important for the signaling complex formation and invadopodia function. Indeed, cholesterol depletion almost completely blocked invadopodia proteolysis, in line with previous reports [67,69]. We propose that lipid rafts in the invadopodial compartment constitute the platform where these interactions occur and are responsible for a regulated ECM proteolytic activity.

Lastly, we used a set of ezrin and NHE1 mutants to dissect the role and possible mechanism of p-ezrin and PIP2 in regulating invadopodia proteolytic action. We found that, indeed, removal of the possibility to phosphorylate ezrin at T567 (T567A, pd) greatly reduced invadopodial proteolytic activity while, conversely, a mutant where the threonine at position 567 is replaced by a negatively charged aspartate, which mimics a permanent phosphorylation (T567D, pmim) resulted in a greatly increased proteolytic capacity of the invadopodia. Importantly, incubation with a pharmacological inhibitor of ezrin phosphorylation at T567 (NSC668394) also reduced invadopodia activity, suggesting that the observed reduction in invasion and metastases in mice treated with NSC [75] is due to a reduced invadopodia activity. The activation of ezrin can also occur through its binding to PIP2, a lipid that is selectively concentrated at the apical surface of polarized epithelia [57,58] and which is also expressed in invadopodia [76]. Indeed, transfection of cells with the ezrin construct in which its binding to PIP2 is abolished (PIP_2_ **^-^**) significantly reduced the total ECM proteolytic capacity and invasion to levels similar to the phospho-dead (pd) mutant. Moreover, in cells transfected with double mutants for phosphorylation and PIP2 binding, the double negative mutant construct (PIP_2_ **^-^**/pd) did not further reduce ECM proteolytic invadopodial activity, whereas co-transfection of the PIP2 negative ezrin mutant construct together with the phosphorylation mimic (PIP_2_^-^ & pmim) construct annulled the effect of the two mutations on both invadopodia proteolytic activity and invasion. It is well known that ezrin is activated by a sequential binding to PIP2 followed by T567 phosphorylation [57,58] and our data show that the binding of ezrin with membrane PIP2 is also a prerequisite for its regulation of invadopodial activity.

The hypothesis that invadopodia p-ezrin overexpression influences their activity in breast cancer cells, at least in part, through the regulation of the NHE1 is further supported by the similar regulatory pattern of invadopodia ECM proteolysis with NHE1 mutants that cannot bind either ezrin or PIP2 (Figure 6B) as was observed above with the ezrin mutants. Indeed, while over-expression of the wild-type (WT) NHE1 construct increased focal digestion by approximately 45%, cells transfected with either the ezrin binding-defective mutant (KRA 556-564) or the PIP2 binding-defective mutant (KRA 513-520) of NHE1 had a greatly reduced amount of focal Matrigel digestion compared to cells transfected with the empty vector. While this inhibition is lower than that reported in cells transfected with the ion transport-defective mutant (E266I) of NHE1 [9], it does compare closely with the amount of inhibition observed above with the ezrin mutants.

Interestingly, the activity of NHE1 can also be directly regulated by its binding to PIP2 suggesting that a versatile p-ezrin-PIP2-NHE1 complex exists where regulation of NHE1 activity could occur through a dynamic interaction of the two pathways and introduces an additional layer of regulation of invadopodia activity between the cytoskeleton and the membrane. Indeed, the localization of the signaling complex in invadopodia lipid rafts could possibly increase the local PIP2 concentration creating not only a protein-protein signalsome complex but a dynamic protein-lipid-protein signalsome complex connected to the actin cytoskeleton that is able to modulate rapidly and precisely the cellular response to changing conditions. NHE1 is targeted by hypoxia [77] and hypoxia was found to stimulate invadopodial localized NHE1 [11] and, therefore, in this way NHE1 constitutes an important point of integration between the interaction of the tumor cell with the ECM through integrin receptors and the metabolic tumor microenvironment and could also be important for the design of novel anti-metastasis therapies.

The mechanism by which p-ezrin stimulates the invadopodial NHE1 is poorly known at present. Signaling modules are orchestrated by scaffolding proteins and a growing body of evidence now indicates that one such scaffolding protein, NHERF1, through its direct binding to ezrin plays an important role in modulating its function. The active, open form of ezrin (p-ezrin) colocalizes with NHERF1 at or near the plasma membrane of polarized cells where they reciprocally stabilize each other and function together in organizing macromolecular complexes [15,16]. Our identification here of p-ezrin as a crucial molecule in breast cancer dissemination suggests that the roles of NHERF1 [78] and NHE1 [78,79] in directing metastasis may well be governed by p-ezrin. Indeed, we find here that it is a phosphorylated form of NHERF1 that binds to the ß1-integrin/p-ezrin/NHE1 complex that forms in the lipid rafts of the invadopodia to finely regulate the development and function of the invadopodia, by selecting and eventually integrating the various signals arriving at the cell. Intriguingly, a similar polarized distribution and interaction of p-ezrin and p-NHERF1 have been shown to be fundamental components of the development of microvilli in a number of epithelial cell types [20-22]. The present data suggest that microvillar growth and development may have profound similarities to the tumor invasive structure and that the trans-differentiation represented by invadopodia in aggressive cells may represent the subversion of the normal microvillar machinery by the invasive tumor cell.

We hypothesize that the protein reorganization in the invadopodia occurs, in part, through binding of phosphorylated NHERF1 with still unidentified sub-membrane proteins. It has been reported that phosphatidyl inositol-4-phosphate 5-kinase (PIP5K), which regulates PIP2 formation [76], interacts with NHERF1 [80] and that active RhoA-GTP affects ezrin activation by the involvement of its effectors Rho Kinase (ROCK) and/or PIP5K which, in turn, regulates PIP2 formation [81]. We have previously described a novel signal transduction module in breast cancer cells localized to the dominant leading edge pseudopodia that, during hypoxia or serum deprivation, increases invasive ability in human breast cancer cells by a stimulation of the NHE1 via the NHERF1 directed and PKA-mediated phosphorylation of RhoA at serine 188 and the subsequent inhibition of RhoA and p38 activities [82,83]. We hypothesize that the increased phosphorylation and altered distribution of ezrin and its binding to phosphorylated NHERF1 during exposure to the ECM promotes invadopodia formation and function and eventual invasion in that this signalsome re-directs PKA to RhoA, which may include a RhoA-ROCK-p38 signal pathway that normally represses NHE1. The release of this repression results in an increase in NHE1 activity and in subsequent invasion. Accordingly, we favor the view that the ß1-integrin receptor binding to the ECM directly facilitates the assembly of a p-ezrin/p-NHERF1 protein complex that probably includes the targeting of PKA to still undescribed sub-membrane proteins to regulate NHE1 activity and subsequent invadopodia formation and activity. Current studies in our laboratory are addressing these interesting and potentially important associations.

Invadopodia assembly is promoted by ECM signaling and collaborations between integrins, the hyaluronan receptor CD44 and growth factor receptors are probably the prerequisite events for their formation and development. There is some uncertainity concerning the relative importance and interaction dynamics of the different ECM and growth factor receptors in invadopodia initiation and development in that it is not yet clear if the basic signal is integrin binding with followed by cross-talk and/or transactivation with the other receptors. Here, we observed that seeding the cells on crosslinked gelatin or on matrigel stimulated the formation of invadopodia containing ß1 integrin and EGF receptors while CD44 was localized to the membranes outside of the invadopodia. This suggests that CD44 is not constituitively expressed in invadopodia but may be recruited there only upon a sufficiently high concentration of its agonist, hyaluronan, which can be concentrated at the invasive front of tumors and may be important in cancer stem cell-niche interactions [84]. As CD44 is directly associated with adhesive podosomes [85], this presence outside of the invadopodia could constitute the mechanism for general adhesion of the cells to the Matrigel. Interestingly, the EGFR was constituitively expressed in invadopodia even in the absence of EGF and this would create an efficient mechanism to permit the cells to rapidly increase invasive capacity upon EGF becoming available. This ß1 integrin-mediated EGFR recruitment and transactivation has been often reported in cancer cells and suggested to be important for a concerted regulation of invasion [86-88] (Morello et al., 2011; Morozevich et al., 2012; Lau et al., 2012) but, here, we show for the first time that this can occur inside ß1 integrin promoted invadopodia.

In this study we aimed at elucidating the role of the phosphorylation of ezrin and its binding to PIP2 and their possible synergism with respect to activation of ezrin’s orchestration of the multiprotein complex in the invadopodia and invadopodia activity. Altogether these results suggest that both the activation of ezrin and its interaction with PIP_2_ are indispensable in controlling invadopodial NHE1 activity and the consequent focal proteolytic capacity. Furthermore, the stimulation of cancer cells to develop invadopodia by plating on ECM, occurs through the formation of a a lipid raft compartmentalized functional protein-lipid-protein “signalsome” formed by NHE1, p-ezrin, PIP2, ß1-integrin and p-NHERF1 that promotes this activation of NHE1. The present study adds ezrin in its T567 phosphorylated state as an important player in the race for understanding the molecular mechanisms behind the cancer invasive process. We believe that p-ezrin could serve as a marker potentially applicable to the detection and identification of pre-symptomatic cancers and, secondly, could be exploited as a therapeutic target in those cancers.

## Materials and Methods

### Patients

Surgical specimens of breast cancer were obtained from 75 consecutive patients with a first diagnosis of primary breast cancer histologically confirmed at the Department of Pathology, Anatomic Pathology A Unit, Istituto Nazionale Tumori, Milan, Italy. Before undergoing routine surgery, all patients signed an informed consent authorizing the Institute to utilize their removed biological tissues for research purposes. Approval was obtained from the Institutional Ethics Board (Dr. Roberto Satolli, president, email: segreteriaCEI@istitutotumori.mi.it) of the National Institute for the Study and Cure of Tumors (IRCCS) of Milano, Italy and is in full agreement with the principles of the Declaration of Helsinki. Routine staging procedures were adopted for determination of stage disease extension according to UICC criteria [89]. The patients underwent surgery before receiving any therapy. Just after surgical removal of biological tissues, the pathologist selected from the primary tumor and from contiguous macroscopically not involved breast tissues samples destined to routine diagnostic practice and to research activities. The characteristics of these tissue samples have been successively confirmed by H&E histological analysis. The cytohistological tumor differentiation grade and hormone receptors (Estrogen Receptor, ER, and Progesterone Receptor, PgR) expression were determined by immunohistochemical assays and categorized as positive or negative cases according to the cut-off value of 10% of positive immunostained cells. Estrogen and Progesterone and their receptors play important roles in the genesis and malignant progression of breast cancer; they are the prototype predictive markers in oncology. Tumor proliferative activity was determined as the percentage of tumor cells expressing the growth-related Ki67 antigen by immunohistochemical assay. The Nottingham Prognostic Index (NPI) combining tumor size, lymph node stage and histological grade information was utilized to score each patient in which patients with NPI values < 2.5 have an expected best prognosis, with NPI = 2.5–3.5 have an expected intermediate prognosis and with NPI > 4.5 are associated with poorest prognosis [60].

### Immunohistofluorescence

Immunohistofluorescence studies were performed on formalin-fixed tissue sections embedded in paraffin wax. The breast cancer tissues, obtained from patient breast biopsy specimens, were fixed in 20% neutral buffered formalin for 24 hours and embedded in paraffin. Serial sections of 3 micron in thickness were obtained from tissue blocks, deparaffinized with xylene and rehydrated in an ethanol series. Microwave pretreatment (slides were immersed in a 10 mM citrated buffer, pH 6.0, at 95°C, 15 min total) for antigen retrieval was carried out prior to incubation with primary antibody. After cooling, slides were washed in distilled water, treated 10 min with 0.2% BSA to block nonspecific protein binding, washed in water for 5 min and then incubated with p-ezrin (1μg/100μl) and/or HER2 (monoclonal H1alpha67, 1:300, Abcam, Cambridge, UK) antibody overnight at 4°C in a humidified chamber. Positive tissue controls of the breast carcinoma as well as negative control slides that were run simultaneously were used to assess the quality of immunostaining. For negative controls, slide sections that were positive for staining were treated with 0.2% BSA instead of the primary antibody. No staining was observed in any of these controls. Images were obtained on a BX40 microscope (Olympus) with a SenSys 1401E-Photometrics CCD camera.

### Statistical Procedures

In the clinical measurements, Ezrin and p-Ezrin expression were quantified by two independent observers and evaluated in each case as negative or positive, where the positivity was assigned when ≥ 30% of the tumor cells expressed Ezrin and/or p-Ezrin. Each case was scored as with cytoplasmic Ezrin localization vs. cell membrane localization, when ≥ 30% of the tumor cells expressed Ezrin in the cytoplasm or in the cell membrane. Each case was classified as cytoplasmic Ezrin localization vs. cell membrane localization, where the result of a trial only determines whether or not the specified event has occurred (cytoplasmic/membrane localization). Modelling using a binomial distribution gave the empirical estimate is the maximum likelihood estimate. The binomial distribution is the discrete probability distribution of the number of successes in a sequence of *n* independent yes/no experiments, each of which yields success with probability *p*. The empirical probability (ratio of the number of outcomes in which a specified event occurs to the total number of trials) is an estimator of a probability, while the standard deviation of a binomial distribution can be calculated as σ = (n*p*(1-p))^1/2^. Kruskal-Wallis non-parametric ANOVA test was applied to analyze ezrin/p-ezrin expression between different grades and NPI stages while the Mann-Whitney non-parametric test was applied to normal and tumor tissues, age, menopausal status, size, node status and the positive vs negative classes of PgR and Ki67. The negative classes were defined as tumors having values below 20 in IHC analysis. Correlation analysis for ezrin/p-ezrin expression and PgR, Ki67 and HER2 expression were performed with the Spearman-Rank non-parametric test.

In the *in vitro* experiments, student’s t-test was applied to analyze the statistical significance between treatments in which P < 0.05 was considered as significant. All comparisons were performed with InStat (GraphPad Software).

### Cell culture and transfection of constructs

MDA-MB-231 cells were cultured as previously described [83]. The following mutated ezrin constructs were made to better dissect the relative importance of its phosphorylation and PIP2 binding capacity on invadopodia proteolytic function. The T567 domain was mutated to either no longer be able to be phosphorylated (T567A) or to mimic phosphorylation (T567D) while it was mutated in the in the PI(4,5)P_2_ (PIP2) binding domain (PIP_2_ **^-^**) to block this capacity. Briefly, we used full-length human ezrin cloned into pEGFP-N1 and mutated at threonine 567 by site-directed mutagenesis [90,91]. Mutations in the PIP2 binding region of ezrin [92] were introduced by substituting four lysines (positions 253, 254, 262 and 263) with four asparagines via site-directed mutagenesis using the following primers: CAGGAACATCTCTTTCAATGACaataatTTTGTCATTAAACCCATCGACAAG (K253N/K254N) and GTTTGTCATTAAACCCATCGACaataatGCACCTGACTTTGTGTTTTATG (K262N/K253N). Mutations were generated using the QuikChange II Site-Directed Mutagenesis kit (Agilent Technologies, Santa Clara, CA) according to manufacturer’s instructions. To produce the myc-tagged version of ezrin and ezrin mutants, the pEGFP-N1 vector (Clontech, Mountain View, CA) was digested with AgeI and BsrGI and religated with myc-containing oligonucleotide adaptor. All constructs were sequence-verified. The phospho(T567) ezrin inhibitor, NSC668394 (NSC), was purchased from EMD Millipore.

Further, a wild-type (WT) NHE1 construct (WT-NHE1-HA) and NHE1 constructs mutated in the PIP2 (KR/A 513-520-NHE1-HA) or ezrin (KR/A 556-564-NHE1-HA) binding domains to no longer bind in those regions were generously supplied by Prof. J. Orlowski of the University of Toranto, Canada.

For transfection, 10 µg plasmid cDNA or empty vector was incubated with 100 µl of LipoTaxi Transfection Reagent (Stratagene, USA) and applied to cells as per manufacturers instructions.

### Matrigel^@^ layer preparation and invadopodia activity assay using *in situ* zymography

For *in vitro* dequenching assay, experiments were conducted in Matrigel™ (diluted to a final concentation of 4mg/ml) containing quenched BODIPYs linked to BSA (DQ-Green BSA) as described [9]. Focal proteolysis produces fluorescence in a black background which is used both to quantify proteolytic activity levels and in co-localization analysis. ‘Pericellular’ digestion is defined as that low level of digestion around the cell that is outside of the strong, focal digestion characterized by invadopodia activity. We obtain this by measuring the level (pixel density) of the release of fluorescence underneight the entire cell while the level of focal digestion/proteolysis is obtained by selecting each focal proteolytic point in each cell and measuring the pixel density. As in the traditional method, each cell can have more than one focal point of digestion. Therefore, the mean pixel density for each cell is the sum of all those contained that cell. There is always a certain number of cells that are negative for focal invadopodial digestion (again as in the traditional technique). The quantity of invadopodia activity was determined with the following measurements: (i) percent of cells with active invadopodia, (ii) number of invadopodia per active cell, (iii) pixal density of digestion performed by individual invadopodia and from these measures the mean total actual invadopodia proteolytic activity for 100 cells was then calculated: Invadopodia Index = percentage of Invadopodia-positive cells (proteolitically-active areas also positive for both actin/cortactin) x mean number of invadopodia/cell.

### Cross-linked gelatin layer preparation and invadopodia fractionation

Porcine skin gelatin was dissolved to a final concentration of 2mg/ml in PBS containing 2mg/ml of sucrose by a slight warming in a microwave oven and 15 ml of this gelatin solution, kept warm at 40°C, was spread on 150mm diameter plastic dishes to evenly cover the entire dish surface. Excess gelatin was removed and the remaining layer was allowed to gel on ice for 10 min. Then 10 ml of ice cold, 0.5% glutaraldhyde in PBS was added to each dish to cross-link the gelatin on ice for 15 min with gentle shaking. The cross-linked gelatin was then washed three times with PBS for 5 min and 20 ml of 70% ethanol was added to each dish for 1hr under a sterile bench hood to sterilize. The gelatin layer was washed two times with sterile PBS for 5 min and two times with complete DMEM, the last wash of DMEM was not removed and the dishes were left in a humified, 37°C incubator for 1hr followed by seeding 4,000,000 cells on each dish. After 24 hr in a humified, 37°C, 5% CO_2_ incubator, cell fractions were isolated as follows: each dish was washed three times with PBS containing 1mM CaCl_2_ and 0.5mM MgCl_2_, two times with 0.2 X PBS plus 1mM CaCl_2_ and 0.5mM MgCl_2_. Cells were then incubated on ice with 3ml of hypertonic swelling buffer (0.2 X PBS supplied with 2μl/ml protease inhibitor cocktail (Sigma), PMSF 1mM, sodium orthovanadate 1mM) for 15 min on ice. Cell bodies were gently scraped with an L-shaped pasteur and centrifuged at 10,300 X g for 30 min. Supernatant was collected (cytosolic soluble proteins) and placed on ice while the pellet was resuspended with 100μl of lysis buffer (Hepes 5mM, EDTA 0.5mM, pH7.2 supplied with protease inhibitor 2μl/ml, PMSF 1mM, sodium orthovanadate 1mM, DTT 1mM, nonidet 0.1%) and membrane proteins were extracted by 30 min of rotating at 4°C. After two washes with PBS, the entire gelatin layer containing entrapped invadopodia was scraped from the dish with 1ml of above lysis buffer. The collected gelatin was vortexed and protein extracted for 30 min on an orbiting wheel at 4°C. The fractions containing membrane proteins or invadopodia were collected in eppendorf tubes and centrifuged at 13,000 X rpm at 4°C. The supernatant of each fraction was collected and the pellet discarded. The diluted samples were concentrated by centrifugation at 10,300 X g for 1hr and protein concentration of the three fractions were measured with Bradford (Pierce). For Western Blotting, proteins were resuspended in SDS sample buffer (6.25 mM Tris-HCl, pH 6.8, containing 10% glycerol).

### Invasion across Matrigel^@^ layer in Boyden Chambers

A quantitative measure of the degree of *in vitro* invasion of MDA-MB-231 cells was measured as the ability to traverse a 8 µm polycarbonate membrane coated with 5 µg matrigel (Chemicon Int., Livermore, CA) as previously described [82]. The fluorescent samples were read in a fluorescence plate reader at 480/520 nm (Cary Eclipse Fluorescence Spectrophotometer, Varian). Mean ± S.E., n=5, ***p<0.001 and n.s. is not significant.

### Co-immunoprecipitation

After treatment, fractions were prepared in ice-cold co-immunoprecipitation lysis buffer (50 mM Tris [pH 7.5], 150 mM NaCl, 1% NP-40, 0.5% sodium deoxycholate, 100 µM Na _3_VO_4_, 1 mM NaF, protease inhibitors). An aliquot was removed for the determination of total cellular protein. Approximately 300 µg protein of the membrane or invadopodia fractions was incubated for 1 hour at 4°C on a rotator with 1µg of primary antibody. 5µl of resuspended volume of Protein A/G PLUS-Agarose (Santa Cruz Biotechnology, CA) was then incubated at 4°C on a rotator overnight. Immunoprecipitates were collected by centrifugation at 2,500 rpm for 5 minutes at 4°C. Supernatant was carefully aspirated and discarded and the pellet washed 4 times with 1 ml lysis buffer, each time repeating centrifugation step above. After the final wash, the pellet was resuspended in 40 µl of sodium dodecyl sulfate (SDS) sample buffer (6.25 mM Tris-HCl, pH 6.8, containing 10% (v/v) glycerol, 3mM SDS, 1% (v/v) 2-mercaptoethanol and 0.75 mM of Bromophenol Blue) and was run on 10% SDS-PAGE, blotted to ImmobilonP and analyzed by Western Blotting with polyclonal p-ezrin antibodies diluted 1:2000 and/or HER2 (monoclonal H1alpha67, 1:300, Abcam, Cambridge, UK)

### Immunofluorescence

Cells plated on coverslips or colonies on matrigel were washed 2 times in sterile PBS at room-temperature, fixed with 3.7% ice-cold paraformaldehyde/PBS for 20 min. The fixed cover-slips were washed 3 times for 5 min each with ice-cold PBS, the colonies permeabilized with 0.1% TRITON X-100 for 10 min and then saturated with 0.1% gelatin in PBS for 10 min. The coverslips were incubated with polyclonal anti-NHERF1 primary antibody (Affinity BioReagents, Golden, CO) diluted 1:300 in 0.1% gelatin in PBS at RT for 1 hr, washed 3 times for 5 min each with 0.1% gelatin in PBS and incubated at RT for 1 hr with the ALEXA 488 goat anti-rabbit IgG secondary antibody conjugate (Molecular Probes, Eugene, OR). The coverslips were then washed 3 times with ice-cold PBS for 5 min each, rapidly rinsed in dH_2_O and then mounted with Mowiol (CalBioChem, San Diego, CA). Proteins were detected with either a Nikon TE 2000S epifluorescence microscope equipped with a MicroMax 512BFT CCD camera (Princeton Instruments, NJ) using a Nikon lamp shutter with a mercury short arc photo optic HBO 103 W/2 lamp for excitation (OSRAM GmbH, Augsburg, Germany). In co-localization experiments, cells were observed at 600X magnification in oil immersion with a laser scanning confocal microscope (LSCM) (C1/TE2000-U; Nikon Instruments SpA, Sesto Fiorentino–FI, Italy) equipped with He/Ne 633 and Argon 488 lasers with 495–519 (B2-A) and 642–660 (Cy5) nm excitation filters. All images were taken under Plan Apo 60XA/1.40 NA oil objective (Nikon, Japan). For each cell or tissue section, scanning was conducted with 25-30 optical series from the top to the bottom of the cell with a step size of 0.45 µm. Parameters related to fluorescence intensity were maintained at constant values in all measurements.

### Image analysis

For every image a Z-stack was acquired using the Metamorph software (Universal Imaging Corp, West Chester, PA) and every three color stack (red, green and blue) is the sum of 3 stacks (one for each color) acquired separately in black and white (B/W). Before image analysis, each stack is deconvolved using the AutoDeblur 9.1 function of the AutoQuant software (Troy, N.Y.) and then merged by transforming the three channels corresponding to red, green and blue into a single two color stack using the ‘RGB merge’ command of the ImageJ^©^ software. To verify colocalization, the three separate B/W stacks were analyzed with the ‘colocalization’ plugin of ImageJ with a ratio of 97, threshold of 50 for both channel 1 and 2. By then selecting the ‘colocalized points (8 bit)’ option a new stack is obtained where the colocalized pixels appear white on a black background. This stack is then converted into a voxel-gradient (VG) shading function of AutoVisualize^©^ (AutoQuant software, Troy, N.Y) which permits the observation of the 3D colocalization zones in a volume.

The random or co-dependent nature of the above calculated ‘apparent’ dye-overlap colocalizations were tested using (i) Intensity Correlation Analysis (ICA) in which the distribution of the intensity value (normalized to 1) for each pixel of a channel is plotted against the the product of the difference of the mean (PDM) of the two channels are determined and (ii) the Li Intensity correlation quotient (ICQ) calculated [93]. Both ICA and ICQ were calculated using the JACoP image analysis package plugin in ImageJ. Apparent colocalization due to random staining or very high intensities in one window will have an ICQ near zero while if the two intensities are interdependent (colocalized) the values will be positive with a maximum of 0.5. In addition to giving information on cellular localization of the proteins, the ICA-ICQ analysis is useful for identifying potential low-affinity interactions within protein complexes that may be missed in high-affinity co-immunoprecipation or pull-down experiments.

### Isolation of Raft and Nonraft fractions

Raft (pellet) and nonraft (supernatant, Supernat) fractions were separated from the above membrane and invadopodia fractions by incubating the fractions (100 µg protein) at 4°C with lysis buffer containing 1% of Lubrol. Insoluble material was formed into pellets (by centrifugation at 100,000 X *g* for 1 hour), and equal amounts of the resuspended pellet (P) and the supernatant (SN) were analyzed by SDS-PAGE and Western blotting.

### Cholesterol depletion

Membrane cholesterol was depleted, as previously described [94], by suspending cells in extracellular solution containing 1 mM Methyl-β-cyclodextrin (MβCD) for 1 hr at 37 °C. For ECM proteolysis imaging experiments, transduced cells were incubated in the DMEM-based culture medium containing 1 mM MβCD for 1hr at 37°C. MβCD treatment significantly reduced the membrane cholesterol content as detected by staining with the fluorescent polyene antibiotic filipin (Sigma-Aldrich) that forms complexes with cholesterol (Figure S4). Control or MβCD-treated cells were fixed in 3.7% formaldehyde in phosphate-buffered saline (PBS) for 10–15 min. After rinsing, cells were incubated with PBS containing 0.05 mg/ml filipin (Sigma-Aldrich) and 10% fetal bovine serum. Fluorescence images were obtained using a D350/50x excitation filter, E420LP dichroic mirror, and a 400 DCLP emission filter.

## Supporting Information

Figure S1 **Activation of ß1 integrin stimulates invadopodia ECM proteolysis by inducing phosphorylation of ezrin at T567.** A. To determine the effect of specific ß1 integrin activation on p-ezrin levels, MDAMB-231 cells were treated with 5mg/ml of the activating antibody P4G11 (Santa Cruz Biotechnologies, sc-18845 L) for the indicated times and the levels of p(T567)-ezrin were measured in Western Blot.
B. To examine the role of ezrin T567 phosphorylation in ß1 integrin stimulation of invadopodial-dependent focal digestion of the ECM, MDA-MB-231 cells were treated with 5mg/ml of the activating antibody P4G11 in the absence or presence of 3μM of the inhibitor of ezrin T567 phosphorylation, NSC668394 (NSC). Cells were plated on Matrigel with DQ-Green BSA and, 24 hr later, ECM digestion was analyzed in confocal microscopy for a series of individual cells as described in Methods. Mean ± S.E.M., n=4, ***p<0.001 and *p<0.05 for focal proteolysis compared to the control cells.
(TIF)

Figure S2 **CD44 localization in invadopodia and interaction with pezrin and ECM digestion.** A. To better visualize invadopodial focal digestion and protein localization in Matrigel, we utilized the *in* *situ* zymography technique using the quenched fluorescent substrate, DQ Green-BSA, such that a quantifiable fluorescence is released only upon digestion of the matrix. Cells seeded on Matrigel were allowed to digest the fluorigenic substrate and after fixation cells were assayed in immunofluorescence. Confocal images in axial planes taken at the bottom of the cells (XY) of a typical cell incubated on Matrigel overnight show p-ezrin (blue), CD44 (red) and digestion (green) localization. For the field, XZ zoomed sections of the above representative regions of interest (white box) are shown at the side. As can be seen in green, low levels of basal, diffuse digestion were observed under and around the cells together with more restricted areas of high levels of focal digestion.
Importantly, p-ezrin (blue) and digestion (green) were co-localized in protrusive digestive structures on the ventral cell surface while CD44 (red) is localized to the cell plasma membrane outside of invadopodia (see RGB plot for a typical zone). ICA analysis plots using the JACoP image analysis plugin in ImageJ for CD44/p-ezrin displayed hour-glass curves for the co-localization of CD44 and p-ezrin and for CD44/digestion demonstrating no significant co-localization of CD44 with either digestion or p-ezrin (ICQ = 0.036 ± 0.015; n.s.). Scale bars = 10 μm(XY) and 5 μm (XZ).
B. CD44 binds to p-ezrin and NHE1 primarily in the membrane fraction. MDA-MB- 231 cells were seeded for 24 hr on 2% gelatin and cytosol, cell body membrane and invadopodia fractions were separated as above. Each cell membrane or invadopodia fraction was then immunoprecipitated with anti-NHE1 or anti-p-ezrin and the precipitated immunocomplex was probed for the expression of CD44 by Western Blot. Input for CD44 was measured by Western Blot of the total input with the same antibodies. Representative blot of 4 independent experiments.
(TIF)

Figure S3 **p-ezrin and ß1-integrin receptor co-localize with areas of focal digestion of Matrigel.** A. Confocal images of a typical cell incubated on Matrigel overnight show p-ezrin (blue), ß1-integrin (red) and digestion (green) co-localization in protrusive digestive structures on the ventral cell surface. To the left of the image are two representative XY areas with their RGB profiles showing a very high co-localization of the two proteins with areas of proteolysis while under the image are two representative XZ constructions of proteolytic focal spots with their RGB profiles showing a very high co-localization of the two proteins with areas of proteolysis along the whole invadopodia. Scale bars = 10 μm(XY) and 5 μm (XZ).
B. To further analyze the potential direct association between p(T567)-ezrin and ß1 integrin at proteolytically active invadopodia, we used an *in* *situ* Proximity Ligation Assay (*in* *situ* PLA) (Duolink II Kit; Olink Bioscience, Uppsala, Sweden), which can detect endogenous protein-protein interactions that occur within 40 nm (Soderberg et al., 2008). We combined this with our Matrigel degradation assay, in which cells are plated on a mixture of Matrigel containing DQGreen BSA-Bodipy and a green fluorescent emission staining is indicative of invadopodia driven-ECM digestion. The advantages of PLA are that this technique provides a fluorescent signal (red) only when two target proteins are colocalized, allowing improved sensitivity for establishing endogenous protein-protein interactions and giving *in* *situ* information whether these co-localizations occur in specific intracellular compartments. Indeed, as shown by the red fluorescent staining reported here (upper left panel), p-ezrin associates with ß1 integrin in a large subset of foci of degraded matrix. Interestingly, there are a couple of ‘digestive’ podosome (doughnut shaped structures, white arrows) where proteolysis occurred mostly within the circle. These results demonstrate that some sub-populations of p-ezrin and ß1 integrin closely interact in functionally active invadopodia where they probably both interact with NHE1. Scale bar = 10 μm.
Söderberg et al., Characterizing proteins and their interactions in cells and tissues using the in situ proximity ligation assay. Methods. 2008 45(3):227-232.
(TIF)

Figure S4 **Effect of MβCD treatment on membrane cholesterol content detected by filipin staining.** In order to visualize the efficiency of MβCD treatment to deplete membrane cholesterol content, cells were incubated with the polyene antibiotic filipin which forms multimeric globular complexes with membrane cholesterol that can be visualized by fluorescence microscopy, was used to verify that MβCD treatment effectively depleted membrane cholesterol. Scale bar = 10 μm.
(TIF)
