# Supplementary figures and images for: ß1 Integrin Binding Phosphorylates Ezrin at T567 to Activate a Lipid Raft Signalsome Driving Invadopodia Activity and Invasion
Source: PLoS One. 2013 Sep 24;8(9):e75113. doi: 10.1371/journal.pone.0075113 (PMC3782503; doi:10.1371/journal.pone.0075113)

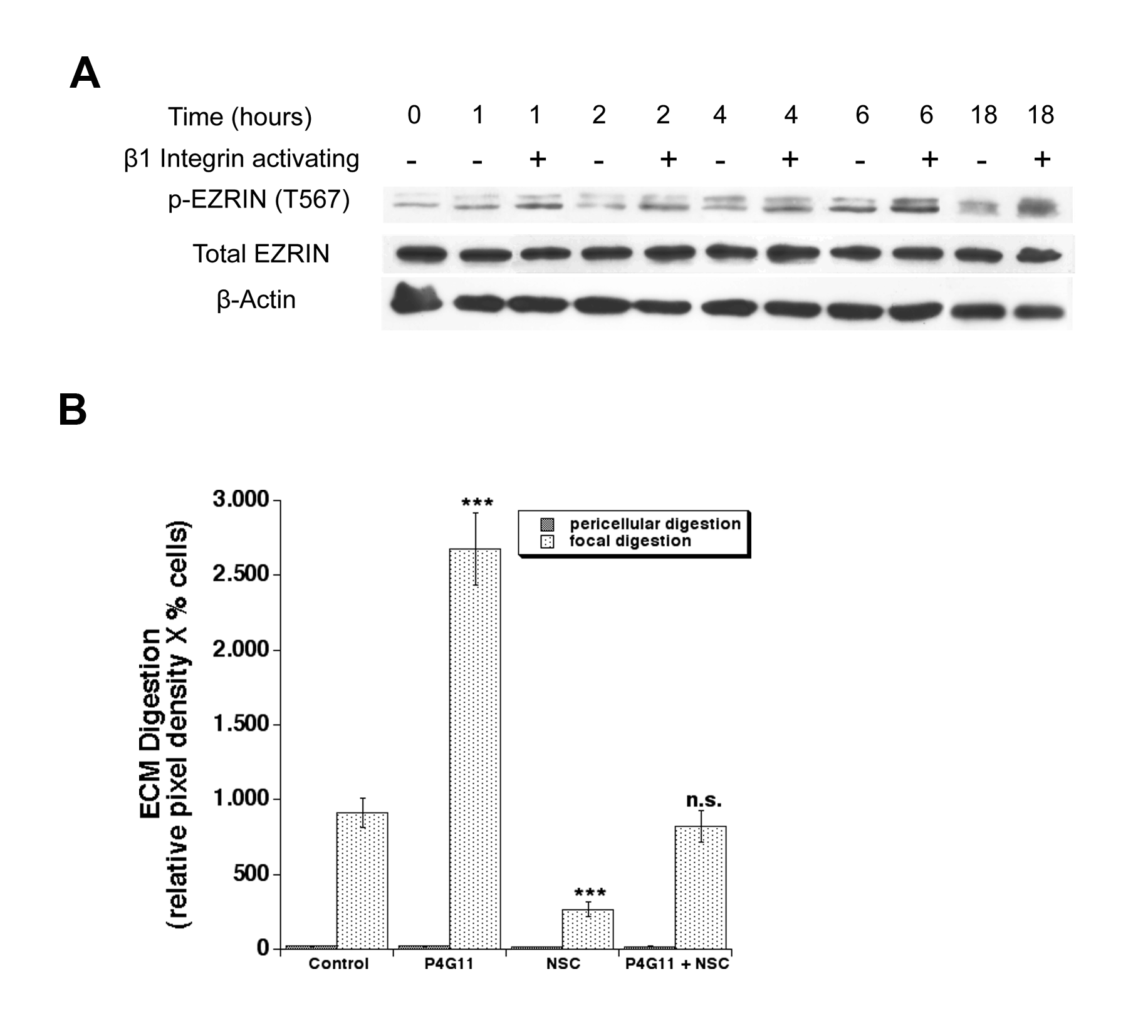

Supplement: Figure S1 — Activation of ß1 integrin stimulates invadopodia ECM proteolysis by inducing phosphorylation of ezrin at T567. A. To determine the effect of specific ß1 integrin activation on p-ezrin levels, MDAMB-231 cells were treated with 5mg/ml of the activating antibody P4G11 (Santa Cruz Biotechnologies, sc-18845 L) for the indicated times and the levels of p(T567)-ezrin were measured in Western Blot. B. To examine the role of ezrin T567 phosphorylation in ß1 integrin stimulation of invadopodial-dependent focal digestion of the ECM, MDA-MB-231 cells were treated with 5mg/ml of the activating antibody P4G11 in the absence or presence of 3μM of the inhibitor of ezrin T567 phosphorylation, NSC668394 (NSC). Cells were plated on Matrigel with DQ-Green BSA and, 24 hr later, ECM digestion was analyzed in confocal microscopy for a series of individual cells as described in Methods. Mean ± S.E.M., n=4, ***p<0.001 and *p<0.05 for focal proteolysis compared to the control cells. (TIF) [file pone.0075113.s001.tif]

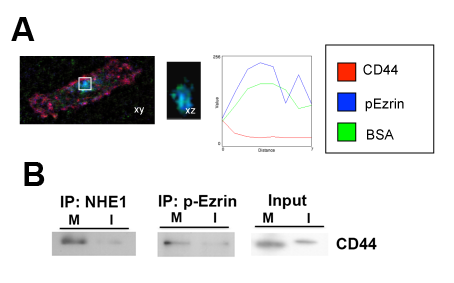

Supplement: Figure S2 — CD44 localization in invadopodia and interaction with pezrin and ECM digestion. A. To better visualize invadopodial focal digestion and protein localization in Matrigel, we utilized the in situ zymography technique using the quenched fluorescent substrate, DQ Green-BSA, such that a quantifiable fluorescence is released only upon digestion of the matrix. Cells seeded on Matrigel were allowed to digest the fluorigenic substrate and after fixation cells were assayed in immunofluorescence. Confocal images in axial planes taken at the bottom of the cells (XY) of a typical cell incubated on Matrigel overnight show p-ezrin (blue), CD44 (red) and digestion (green) localization. For the field, XZ zoomed sections of the above representative regions of interest (white box) are shown at the side. As can be seen in green, low levels of basal, diffuse digestion were observed under and around the cells together with more restricted areas of high levels of focal digestion. Importantly, p-ezrin (blue) and digestion (green) were co-localized in protrusive digestive structures on the ventral cell surface while CD44 (red) is localized to the cell plasma membrane outside of invadopodia (see RGB plot for a typical zone). ICA analysis plots using the JACoP image analysis plugin in ImageJ for CD44/p-ezrin displayed hour-glass curves for the co-localization of CD44 and p-ezrin and for CD44/digestion demonstrating no significant co-localization of CD44 with either digestion or p-ezrin (ICQ = 0.036 ± 0.015; n.s.). Scale bars = 10 μm(XY) and 5 μm (XZ). B. CD44 binds to p-ezrin and NHE1 primarily in the membrane fraction. MDA-MB- 231 cells were seeded for 24 hr on 2% gelatin and cytosol, cell body membrane and invadopodia fractions were separated as above. Each cell membrane or invadopodia fraction was then immunoprecipitated with anti-NHE1 or anti-p-ezrin and the precipitated immunocomplex was probed for the expression of CD44 by Western Blot. Input for CD44 was measured by Western Blot of the [file pone.0075113.s002.tif]

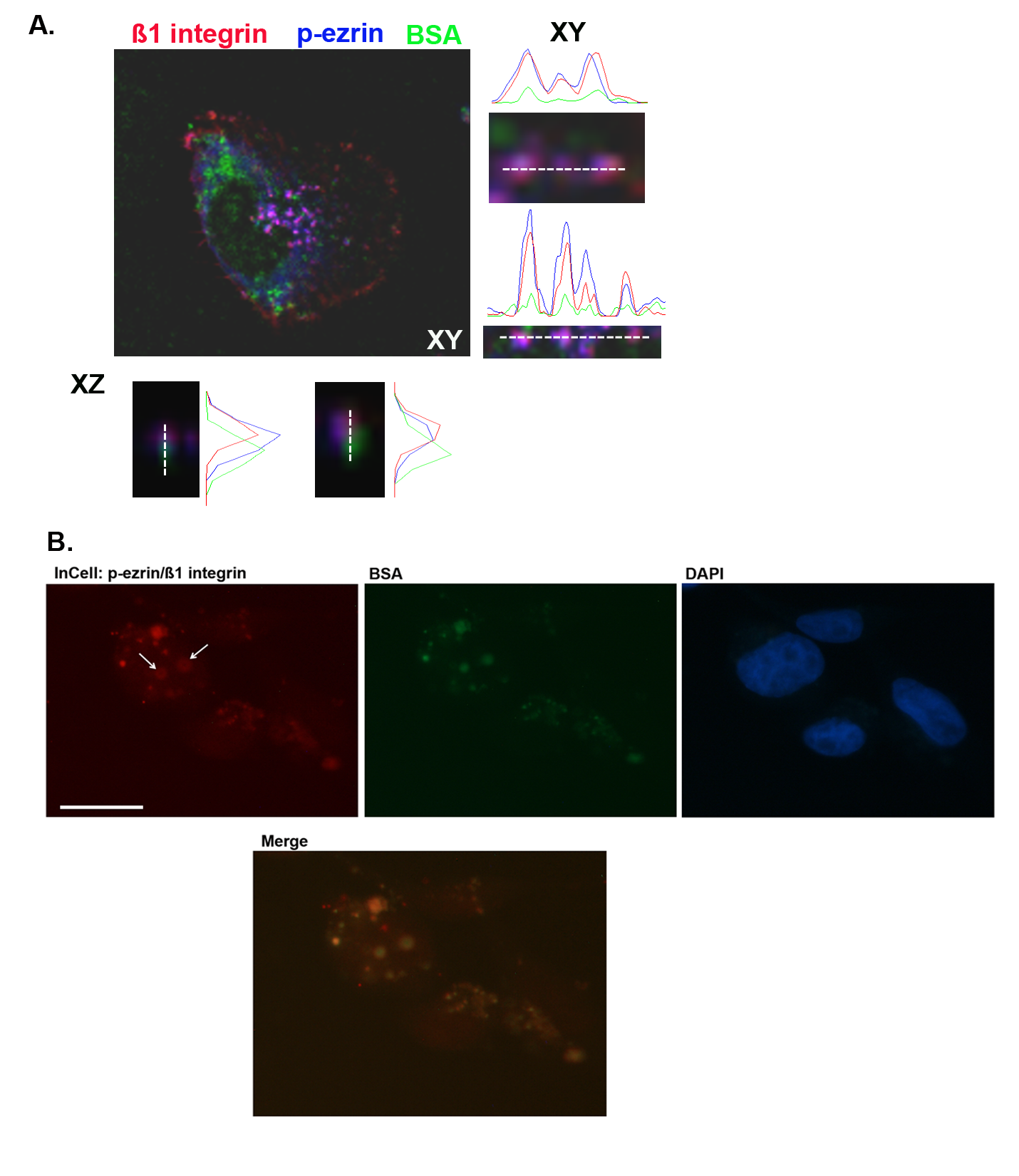

Supplement: Figure S3 — p-ezrin and ß1-integrin receptor co-localize with areas of focal digestion of Matrigel. A. Confocal images of a typical cell incubated on Matrigel overnight show p-ezrin (blue), ß1-integrin (red) and digestion (green) co-localization in protrusive digestive structures on the ventral cell surface. To the left of the image are two representative XY areas with their RGB profiles showing a very high co-localization of the two proteins with areas of proteolysis while under the image are two representative XZ constructions of proteolytic focal spots with their RGB profiles showing a very high co-localization of the two proteins with areas of proteolysis along the whole invadopodia. Scale bars = 10 μm(XY) and 5 μm (XZ). B. To further analyze the potential direct association between p(T567)-ezrin and ß1 integrin at proteolytically active invadopodia, we used an in situ Proximity Ligation Assay (in situ PLA) (Duolink II Kit; Olink Bioscience, Uppsala, Sweden), which can detect endogenous protein-protein interactions that occur within 40 nm (Soderberg et al., 2008). We combined this with our Matrigel degradation assay, in which cells are plated on a mixture of Matrigel containing DQGreen BSA-Bodipy and a green fluorescent emission staining is indicative of invadopodia driven-ECM digestion. The advantages of PLA are that this technique provides a fluorescent signal (red) only when two target proteins are colocalized, allowing improved sensitivity for establishing endogenous protein-protein interactions and giving in situ information whether these co-localizations occur in specific intracellular compartments. Indeed, as shown by the red fluorescent staining reported here (upper left panel), p-ezrin associates with ß1 integrin in a large subset of foci of degraded matrix. Interestingly, there are a couple of ‘digestive’ podosome (doughnut shaped structures, white arrows) where proteolysis occurred mostly within the circle. These results demonstrate that some sub-populations of p [file pone.0075113.s003.tif]

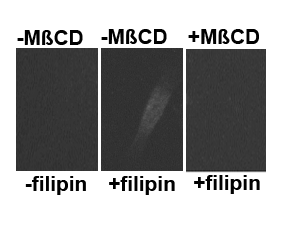

Supplement: Figure S4 — Effect of MβCD treatment on membrane cholesterol content detected by filipin staining. In order to visualize the efficiency of MβCD treatment to deplete membrane cholesterol content, cells were incubated with the polyene antibiotic filipin which forms multimeric globular complexes with membrane cholesterol that can be visualized by fluorescence microscopy, was used to verify that MβCD treatment effectively depleted membrane cholesterol. Scale bar = 10 μm. (TIF) [file pone.0075113.s004.tif]
